# Phytochrome and retrograde signalling pathways converge to antagonistically regulate a light-induced transcriptional network

**DOI:** 10.1038/ncomms11431

**Published:** 2016-05-06

**Authors:** Guiomar Martín, Pablo Leivar, Dolores Ludevid, James M. Tepperman, Peter H. Quail, Elena Monte

**Affiliations:** 1 Department of Molecular Genetics, Center for Research in Agricultural Genomics (CRAG), CSIC-IRTA-UAB-UB, Barcelona 08193, Spain; 2 Department of Bioengineering, IQS School of Engineering, Barcelona 08017, Spain; 3 Department of Plant and Microbial Biology, University of California, Berkeley, California 94720, USA; 4 Plant Gene Expression Center, Agriculture Research Service (ARS), US Department of Agriculture (USDA), Albany, California 94710, USA

## Abstract

Plastid-to-nucleus retrograde signals emitted by dysfunctional chloroplasts impact photomorphogenic development, but the molecular link between retrograde- and photosensory-receptor signalling has remained unclear. Here, we show that the phytochrome and retrograde signalling (RS) pathways converge antagonistically to regulate the expression of the nuclear-encoded transcription factor GLK1, a key regulator of a light-induced transcriptional network central to photomorphogenesis. *GLK1* gene transcription is directly repressed by PHYTOCHROME-INTERACTING FACTOR (PIF)-class bHLH transcription factors in darkness, but light-activated phytochrome reverses this activity, thereby inducing expression. Conversely, we show that retrograde signals repress this induction by a mechanism independent of PIF mediation. Collectively, our data indicate that light at moderate levels acts through the plant's nuclear-localized sensory-photoreceptor system to induce appropriate photomorphogenic development, but at excessive levels, sensed through the separate plastid-localized RS system, acts to suppress such development, thus providing a mechanism for protection against photo-oxidative damage by minimizing the tissue exposure to deleterious radiation.

## Abstract (web summary)

Retrograde signals from dysfunctional chloroplasts influence plant response to light. Here the authors show that the GUN1 retrograde signalling pathway acts antagonistically to the phytochrome-mediated red light perception pathway to control the expression of GLK1, a key transcriptional regulator of photomorphogenesis.

## Graphical abstract

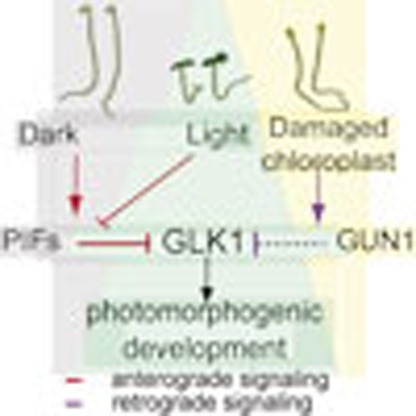

Interorganellar communication coordinates gene expression of nuclear and organelle genomes and is essential to ensure cell fitness. In some eukaryotes, bidirectional communication between mitochondria and nucleus regulates the processes such as life span (yeast), cell cycle (*Drosophila*) or tumour progression (animal cells)^1^. In plants, where the majority of chloroplast components are encoded in the nucleus, anterograde regulation from nucleus to chloroplast and retrograde signalling (RS) from chloroplast to nucleus, are critical to adjust chloroplast biogenesis and photosystem assembly to the prevailing environment and chloroplast status^2^,^3^,^4^. Despite the important implications of RS on cellular function in all organisms, insight into the underlying mechanisms remains sparse.

Deetiolation is the critical switch from skotomorphogenic to photomorphogenic development, triggered in the seedlings emerging from subterranean darkness into sunlight, and is characterized by an extensive transcriptional reprogramming that drives the morphological changes necessary to establish a normal green photosynthetically active seedling^5^. In the dark, skotomorphogenic seedlings use fast-growing hypocotyls to rapidly reach the soil surface, together with a protective apical hook and appressed cotyledons with undeveloped chloroplasts. In contrast, light inhibits hypocotyl elongation and stimulates cotyledon separation and expansion, congruent with the development of functional chloroplasts, thus enabling light capture for photosynthesis. A subgroup of basic helix-loop-helix transcriptional regulators, termed phytochrome-interacting factors(PIFs), accumulate in the dark and promote skotomorphogenesis through regulation of about 10% of the genes in the genome^6^. Light exposure activates the phytochrome family of red and far-red light-responsive photoreceptors that directly interact with the PIFs and trigger their proteolytic degradation, thereby redirecting gene expression to drive deetiolation^7^,^8^,^9^. Indicative of the central role of the PIFs in this process, a dark-grown quadruple *pifq* mutant, deficient in four PIFs (PIF1, PIF3, PIF4 and PIF5), largely phenocopies wild-type (WT) seedlings grown in the light at both the morphological and transcriptional levels. These mutant seedlings display a constitutively photomorphogenic (*cop*-like) phenotype, which includes partially developed chloroplasts^10^,^11^.

On the basis of the phenotypes of plants with induced plastid dysfunctionality, plastid signals have been reported to impact normal light-regulated development^12^,^13^,^14^,^15^. Although the evidence indicates that RS can repress some aspects of early seedling photomorphogenic development, in addition to affecting chloroplast biogenesis^12^,^13^,^14^,^15^, mechanistic insight into the connection between the photomorphogenic and RS pathways has been lacking. While many of the components of the phytochrome pathway (PIFs) and the RS pathway (GUN1, GLK1) examined in the present work have been reported in a number of publications^9^,^10^,^12^,^16^,^17^,^18^, here we present data that connect the two in a comprehensive fashion, identifying the molecular framework that integrates the two pathways at a central node at the apex of a transcriptional network that regulates early seedling photomorphogenic development. We show that the phytochrome and the GUN1 RS pathways act antagonistically to control the expression of *GLK1*, a key transcriptional regulator of photomorphogenesis directly repressed by the PIFs in the dark. Whereas light at moderate levels acts through the phytochrome/PIF sensory-photoreceptor system to induce *GLK1* expression and photomorphogenic development, light at excessive levels is sensed by the plastid and represses *GLK1* induction and photomorphogenesis through a GUN1-mediated RS mechanism independent of PIF mediation. Our data indicate that RS provides a mechanism for protection against photo-oxidative damage by minimizing the tissue exposure to deleterious radiation.

## Results

### Retrograde signals suppress PIF-mediated photomorphogenesis

**Figure 1:**
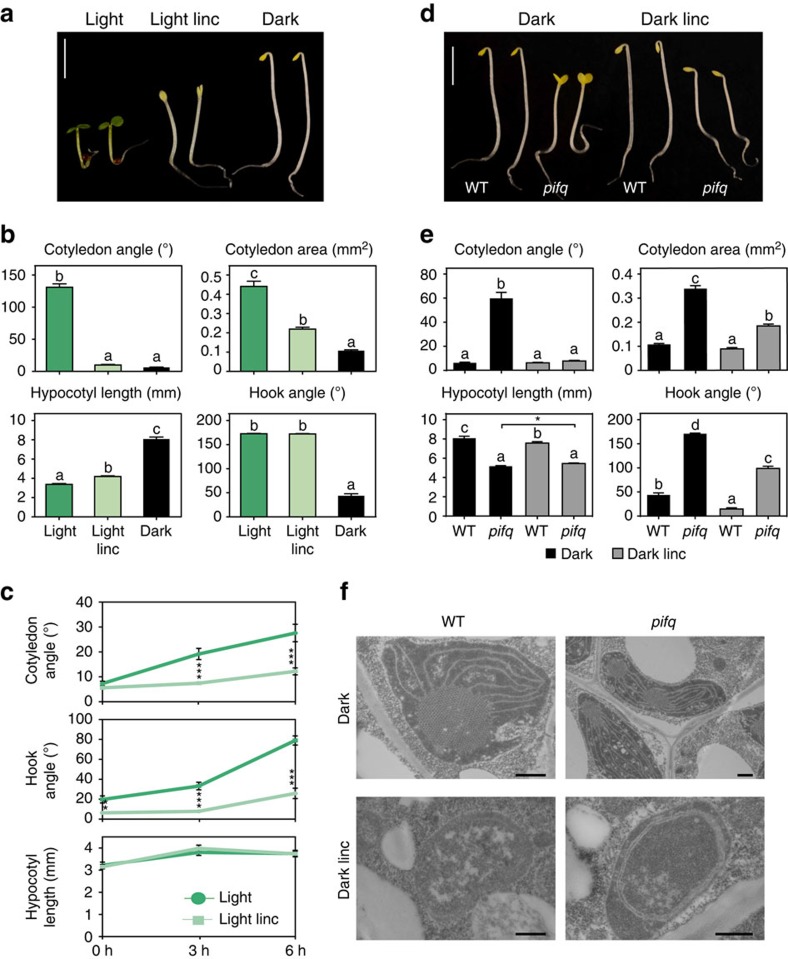
RS from the chloroplast suppresses PIF-mediated seedling photomorphogenesis. (**a**) Lincomycin treatment prevents *Arabidopsis* seedling deetiolation in continuous white light. WT seedlings were grown for 3 days in the light in the absence (Light) or presence (Light linc) of lincomycin. For comparison, 3-day-old dark-grown seedlings are shown at right. Scale bar, 2.5 mm. (**b**) Cotyledon angle and area, hypocotyl length and hook angle of seedlings grown as in a. (**c**) Lincomycin inhibits early deetiolation during the transition of dark-grown seedlings to light. Quantification of cotyledon angle (top), hook angle (middle) and hypocotyl length (bottom) of 2-day-old dark-grown WT seedlings transferred to white light for the indicated times, grown in the absence (dark green) or presence (light green) of lincomycin. (**d**) Lincomycin treatment suppresses the *cop*-like phenotype of *pifq* seedlings in the dark. WT and *pifq* seedlings were grown for 3 days in the dark in the absence (Dark) or presence (Dark linc) of lincomycin. (**e**) Cotyledon angle and area, hypocotyl length and hook angle of seedlings grown as in d. (**f**) Higher-magnification micrographs of samples prepared as in d. Representative etioplasts are shown for WT (left) and *pifq* (right) seedlings grown in absence (top) or presence (bottom) of lincomycin. Scale bar, 500 nm. Error bars in **b**,**c** and **e** represent s.e.m. (*n*⩾20). The experiments were repeated two times with similar results. In **b**,**e** different letters denote statistically significant differences among means by Tukey-b test (*P*<0.05). In **c** and **e**, statistically significant differences between mean values by Student's *t*-test are shown (*P<0.05; **P<0.01 and ***P<0.001).

As an experimental system, we treated light-grown seedlings with lincomycin, a drug known to activate RS by inhibiting chloroplast biogenesis^19^. Strikingly, we found that WT seedlings grown on lincomycin in white light resembled dark-grown seedlings, with long hypocotyls and appressed unexpanded cotyledons that did not accumulate chlorophyll (Fig. 1a,b). This effect was also observed under red light (Supplementary Fig. 1) and after treatment with norflurazon, a herbicide that inhibits carotenoid biosynthesis and causes photo-oxidative damage of chloroplasts in white light^20^,^21^ (Supplementary Fig. 2). The effect of lincomycin on the early development of WT seedlings observed here was considerably stronger than in some previous studies^12^,^22^. This discrepancy may have resulted from these studies having used seedlings grown on 2% sucrose (which we did not administer in our experiments), or from differences in the light conditions. It is well-documented that exogenous sucrose significantly alters normal light-regulated gene-expression and metabolic-pathway patterns, together with associated deviations in growth and developmental responses^23^,^24^,^25^,^26^. On the other hand, our findings are similar to, and extend, other studies showing that defects in chloroplast biogenesis produced by mutations in the SIGMA 2 and 6 components of the chloroplast RNA polymerase (which induce RS similarly to lincomycin)^27^ prevented normal seedling photomorphogenesis under continuous light (Supplementary Fig. 3)^28^. When we examined the kinetics of the deetiolation process, we found that the rapid cotyledon separation response, normally exhibited by dark-grown WT seedlings upon light exposure, was nearly completely blocked in the presence of lincomycin, consistent with the end point analysis (Fig. 1c). We also observed an inhibitory effect on the rapid hook-opening response, indicating that lincomycin has an early effect on hook development. However, this recovered over time and is not detectable in the end point analysis. These observations suggest that RS activity either pre-exists or can be deployed very rapidly in defective plastids, to prevent the normal light-induced response in the seedling apex, very early during deetiolation. In addition, our results indicate that the effects of RS induced by defects in plastid function, are not confined to chloroplast biogenesis, but rather pleiotropically repress seedling photomorphogenesis in the light^15^. This conclusion raises the possibility that the informational-light-induced, PIF-mediated transcriptomic changes that normally implement the photomorphogenic programme, in response to activation of the sensory-photoreceptor pathways, might be affected under these conditions. A previous study showing significant overlap between the transcriptomic changes induced by lincomycin and by a shift from low-to-high fluence-rate white- or blue-light^14^ supports this possibility.

To examine this potential PIF involvement genetically, we analysed *pifq* mutants in the presence of lincomycin. We reasoned that if the PIF-regulated network is also targeted by RS to inhibit photomorphogenesis in the light, we should detect an effect on the photomorphogenic phenotype induced by the absence of the PIF-quartet in the *pifq* mutant in the dark. Strikingly, we found that lincomycin does indeed strongly suppress the *cop*-like phenotype of *pifq* in the dark (Fig. 1d,e; Supplementary Fig. 4). The drug completely suppressed cotyledon separation in *pifq* seedlings, sustained hook curvature, and restored cotyledon appression to levels similar to those exhibited by etiolated WT seedlings in darkness. Lincomycin also induced a modest (1 mm) but statistically significant (Student's *t*-test) increase in hypocotyl elongation in *pifq* seedlings compared with the untreated control (see Fig. 1d,e; Supplementary Fig. 4). Partial suppression of the constitutive photomorphogenic phenotype was also observed in dark-grown *cop1* seedlings in the presence of lincomycin (Supplementary Fig. 5), in agreement with the notion that the constitutively photomorphogenic phenotype of *cop1* in the dark might be caused, in part, by the lower accumulation of PIFs seen in *cop1* (ref. 29), and consistent with previous results in pea^19^. In contrast to lincomycin, norflurazon treatment did not induce RS and did not affect the phenotype of *pifq* in the dark (Supplementary Fig. 6), in agreement with the fact that light is needed to induce RS in carotenoid-deficient seedlings^20^,^21^. In dark-grown WT seedlings, lincomycin did not alter the etiolated phenotype, having only a minor effect on hook curvature (Fig. 1d,e; Supplementary Fig. 4). At the subcellular level, treatment with lincomycin interfered with plastid development in both WT and *pifq* dark-grown seedlings, preventing the partial differentiation of etioplasts into chloroplasts observed in non-treated *pifq* seedlings in the dark (Fig. 1f).

### RS antagonize the light induction of PIF-repressed genes

**Figure 2:**
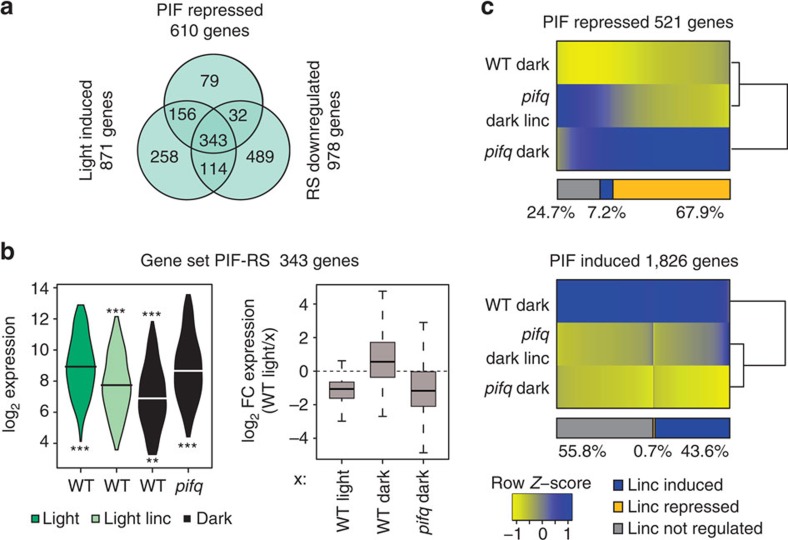
Retrograde signals repress the light-induced expression of PIF-repressed genes. (**a**) Plastid signals repress the induction of light-induced genes. Overlap among genes downregulated by chloroplast RS^27^, induced by red-light^11^ or repressed by PIFs in the dark^11^, identifies 343 genes labelled as ‘Gene-Set PIF-RS' (gene lists provided in Supplementary Data 1). (**b**) Expression of genes in ‘Gene-Set PIF-RS' in WT and *pifq* mutants grown in the light in the absence (dark green) or presence (light green) of lincomycin, or in the dark (black) (left). Fold-change expression of genes in ‘Gene-Set PIF-RS' between WT light lincomycin with respect to WT light, WT dark or *pifq* dark (right). Data obtained from refs 11 and 23. Statistically significant differences from WT light or WT light linc by heteroscedastic *t*-test are indicated in the upper and lower part, respectively (**P<0.01 and ***P<0.001). (**c**) Lincomycin extensively restores the PIF-repressed network in *pifq* in the dark to the WT levels. Two-dimensional-cluster diagram depicting expression levels of 521 PIF-repressed (top) and 1,826 PIF-induced (bottom) SSTF genes in the dark in the absence (dark) or presence (dark linc) of lincomycin. Genes were identified by transcriptomic profiling of seedlings grown for 3 days in the dark in the absence (dark) or presence (dark linc) of lincomycin.

Because defects in chloroplast biogenesis activate RS, and activation of RS is well-known to regulate nuclear gene expression^14^,^23^, we analysed whether RS might indeed be able to target the PIF-regulated transcriptome during early seedling development. For this purpose, we initially used published data^11^,^23^,^27^. Interestingly, of 871 previously defined red light-induced genes^11^, 457 (52%) are inhibited in their normal light responsiveness by RS (in agreement with Ruckle *et al*.^14^), and of those, 343 (75%) correspond to genes shown previously^11^ to be repressed by the PIFs in the dark (Fig. 2a; Supplementary Data 1). We labelled these 343 genes as ‘Gene-Set PIF-RS' (for PIF- and RS-repressed). Moreover, the expression levels of this gene-set in WT seedlings grown in the presence of light and lincomycin resembled those in untreated WT in the dark, indicating that RS is, directly or indirectly, blocking their light responsiveness (Fig. 2b; Supplementary Fig. 7). Importantly, our analysis shows further that this congruent regulation of gene expression by RS and PIFs is strongly selective for light-induced/PIF-repressed genes, because only 19 of the 313 previously identified light-repressed/PIF-induced genes^11^ were re-induced by lincomycin in light-grown WT seedlings, and their expression level was not restored to WT-dark levels (Supplementary Fig. 8). These results suggest that RS inhibits deetiolation, at least partly, by antagonizing the light-induced expression of a large set of PIF-repressed genes, without altering the normal light-triggered repression of the PIF-induced gene set.

Newly performed RNA-seq-based transcriptomic analysis here of dark-grown, non-drug-treated, WT and *pifq* seedlings, identified 521 PIF-repressed and 1,826 PIF-induced genes whose expression changed statistically significantly and by at least twofold (SSTF) in 3-day-old *pifq* seedlings compared with WT in the absence of light (see Supplementary Note 1 for a detailed description of the analysis). Lincomycin strongly, but selectively, reversed this *pifq* molecular phenotype, extensively and preferentially restoring the status of the PIF-repressed network in the *pifq* mutant to WT levels (Fig. 2c; Supplementary Note 1), by statistically significantly repressing the expression of 68% (354) of the PIF-repressed SSTF genes (Supplementary Figs 9 and 10; Supplementary Note 1; Supplementary Data 2). This 354-gene set corresponds predominantly to PIF-repressed/light-induced genes that have been shown in previous reports to be downregulated by RS in the light in WT seedlings^27^, and largely overlaps with the ‘Gene-Set PIF-RS' defined above (Supplementary Note 1; Supplementary Fig. 11). Interestingly, only 9–13% of the genes in these sets are direct PIF targets (Supplementary Fig. 12)^16^, suggesting indirect regulation of the majority by the PIFs. The two largest represented groups correspond to chloroplast- and nuclear-localized proteins (Supplementary Fig. 13). These results show that activation of RS in the dark reverses the expression of the transcriptional network induced in *pifq*, concomitant with the suppression of the *cop*-like seedling phenotype of the mutant. Altogether, our findings indicate that the RS preferentially targets the PIF-repressed genes in restraining light-induced deetiolation. This conclusion is in contrast to a previous model proposing the converse, namely, that integration of the RS and photoreceptor-mediated light signals was accomplished via informational-light induction of genes that were further induced by the RS^14^. Moreover, our findings suggest that the RS and informational-light-regulated signalling pathways must converge downstream of the PIFs to antagonistically regulate the initiation of seedling photomorphogenesis. This conclusion is in contrast to a previous model based on small differences detected in *hy5* mutants, proposing that the RS ‘rewires' the light signalling network in a manner that converts informational light from a positive to a negative signal and vice versa, through transforming HY5 from a positive to a negative regulator of gene expression^13^. Our findings support a model for PIF-regulated genes, in which RS represses the light induction of PIF-repressed genes by a mechanism that acts in the absence of the PIF proteins and, therefore, is independent of any direct RS regulation of PIF transcriptional activity.

### GUN1-mediated RS antagonizes PIF-regulated gene expression

**Figure 3:**
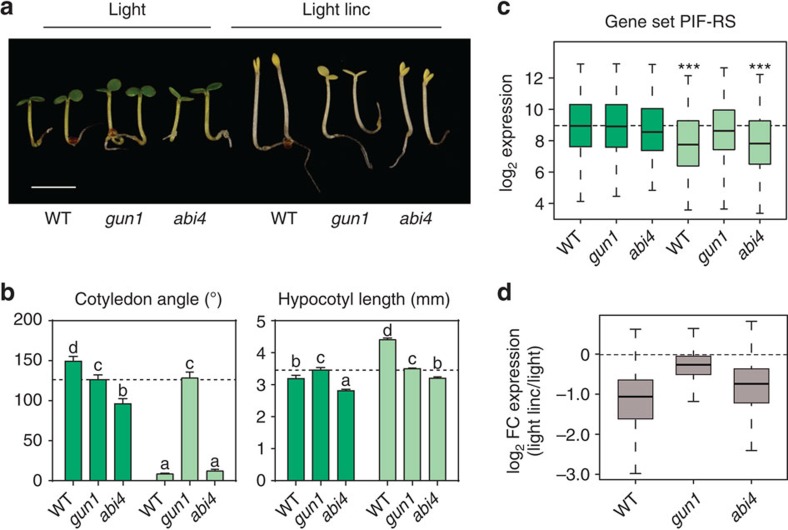
GUN1 mediates the RS that antagonizes PIF-regulated gene expression. (**a**) Mutants in *GUN1* partially deetiolate in the light in the presence of lincomycin, whereas response of mutants in *ABI4* responded more similar to WT. WT, *abi4* and *gun1* seedlings were grown for 3 days in white light in the absence (Light) or presence (Light linc) of lincomycin. Scale bar, 2.5mm. (**b**) Cotyledon angle and hypocotyl length of seedlings grown as in a. Error bars represent the s.e.m. of two independent experiments, each time sampling (*n*⩾25). Different letters denote statistically significant differences among means by Tukey-b test (*P*<0.05). (**c**) Lincomycin-induced repression in gene expression in the light of the 343 genes in ‘Gene-Set PIF-RS' is blocked in *gun1*. Expression of genes in ‘Gene-Set PIF-RS' in WT, *abi4* and *gun1* in the absence (dark green) and presence (light green) of lincomycin. Statistically significant differences from WT light by heteroscedastic *t*-test are indicated (***P<0.001). (**d**) Fold-change expression in the light in the presence (Light linc) with respect to absence (Light) of lincomycin in WT, *abi4* and *gun1* seedlings. Data for **c**,**d** were obtained from ref. 23.

To begin to gain further insight into how the RS pathway might converge on the PIF-regulated transcriptional network, we treated light-grown *abi4*, *gun1*, *gun5* and *gun6* mutant seedlings with lincomycin. GUN1 and ABI4 are two major described mediators of the RS pathway^23^, and GUN5 and GUN6 are involved in plastid-to-nucleus signal transduction^21^,^30^. We found that lincomycin prevented full deetiolation of *abi4*, *gun5*, and *gun6* seedlings in the light, similarly to WT (Fig. 3a,b; Supplementary Figs 14 and 15). However, in striking contrast, *gun1* seedlings partially deetiolated in the presence of lincomycin in the light, displaying light-imposed inhibition of hypocotyl elongation and open and expanded cotyledons (Fig. 3a,b; Supplementary Figs 14 and 15). To examine the molecular phenotypes, we assessed the role of GUN1 and ABI4 in the regulation of the expression of the ‘Gene-Set PIF-RS' defined above. By analysing the available expression data for *gun1* and *abi4* (ref. 23), we found that these genes responded strongly to lincomycin in WT and *abi4*, but not in *gun1*, which displayed gene expression levels in lincomycin-grown seedlings, comparable to those of seedlings not treated with lincomycin (Fig. 3c,d). From these collective data, we conclude that GUN1, but likely not ABI4, GUN5 or GUN6, is necessary for the production, transmission or implementation of the lincomycin-induced RS that inhibits the photoreceptor-mediated, light-induced expression of a subset of light-induced/PIF-repressed genes, thereby repressing seedling photomorphogenesis in the light in response to chloroplast dysfunction.

### PIFs and RS antagonistically regulate *GLK1* transcription

**Figure 4:**
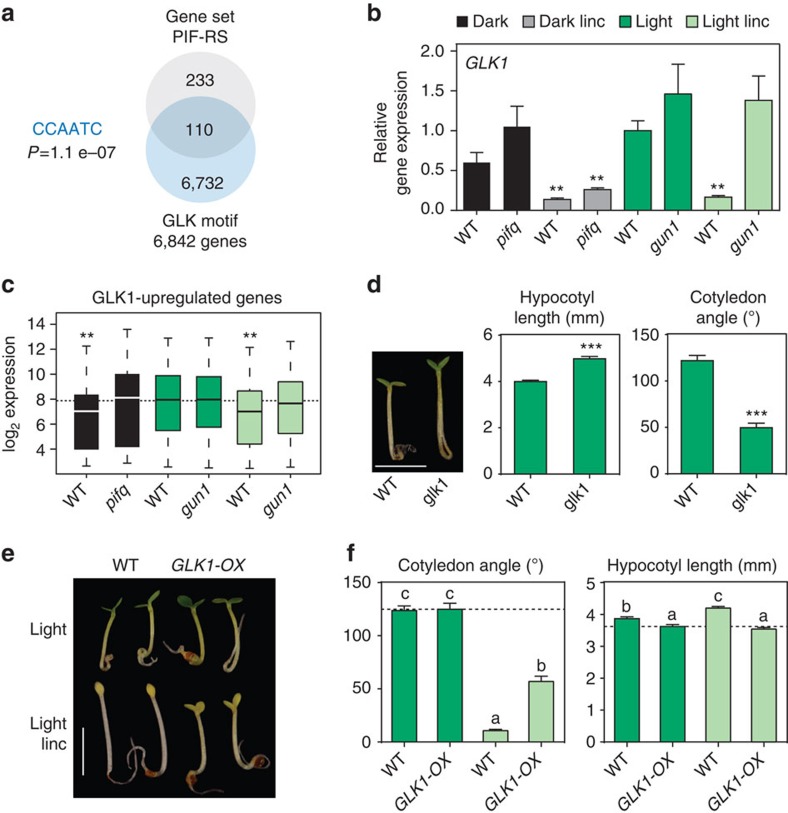
Retrograde and PIF-mediated light signals antagonistically regulate deetiolation through transcriptional control of *GLK1*. (**a**) Promoter regions (500 bp upstream of the start codon) of PIF-RS genes are significantly enriched in putative GLK-binding motifs (CCAATC)^17^, present in 6,842 promoter regions in the *Arabidopsis* genome. (**b**) *GLK1* is a gene whose expression is repressed by the PIFs in the dark and by lincomycin in a GUN1-dependent fashion in the light. Transcript levels of *GLK1* analysed by qRT–PCR in 3-day-old WT, *pifq* and *gun1* seedlings grown in the dark or in continuous white light in the absence (Dark and Light, respectively) or presence (Dark linc and Light linc, respectively) of lincomycin. Values were normalized to *PP2A*, and expression levels are expressed relative to WT light set at one. Data are the means±s.e.m. of biological triplicates (*n*=3). (**c**) GLK1-induced genes display a pattern of expression similar to *GLK1*. Transcript levels were obtained from refs 11 and 23, for a set of 93 genes previously defined as GLK1-induced^17^, which are represented in the ATH1 array. Statistically significant differences from WT Light by heteroscedastic *t*-test are indicated (**P<0.01). (**d**) GLK1 promotes photomorphogenesis under continuous white light. WT and *glk1* mutant seedlings grown for 3 days in the light display a hyposensitive phenotype with partially closed cotyledons and longer hypocotyls. Scale bar, 2.5 mm. (**e**) Seedlings overexpressing *GLK1* display insensitivity to lincomycin and partially deetiolate in the presence of lincomycin. WT and *GLK1-OX* seedlings were grown for 3 days in white light in the absence (Light) or presence (Light linc) of lincomycin. Scale bar, 2.5 mm. (**f**) Cotyledon angle and hypocotyl length of seedlings grown as in e. Letters denote the statistically significant differences among means by Tukey-b test (*P*<0.05). Error bars in **d**,**f** represent s.e.m. of two independent experiments (*n*⩾20). In **b** and **d**, statistically significant differences from light-grown WT seedlings by Student's *t*-test are shown (**P<0.01 and ***P<0.001).

Because the *GUN1* gene does not appear to bind PIFs in its promoter^16^, and is not a PIF-regulated gene (Supplementary Data 2), phytochrome-mediated light- and plastid-RS signalling must converge downstream of both GUN1 and the PIFs, likely through co-regulation of one or more common target genes. Interestingly, analysis of DNA-binding motifs revealed that the ‘Gene-Set PIF-RS' is significantly and specifically enriched in the putative GLK-binding site CCAATC (*z*-score=5.35; Fig. 4a)^17^. *GLK1* is a PIF-repressed gene, which is directly targeted by PIFs in the dark^16^,^31^,^32^ (Supplementary Fig. 16), and that has been proposed to respond to RS downstream of GUN1 (refs 17 and 18). This evidence suggested to us that GLK1 might provide clues to the link between informational-light/PIF and RS/GUN1 signalling. Consistent with this notion, *GLK1* expression is induced in light-grown WT seedlings, as well as being upregulated in *pifq* in the dark^11^,^16^. We found also that the expression induced in both conditions was largely suppressed by lincomycin, and in a GUN1-dependent fashion (at least in the light) extending previous findings^17^ (Fig. 4b). These results indicate that RS acts on or upstream of *GLK1*. Moreover, a similar expression pattern was observed for a set of 93 genes previously defined as GLK1-induced^17^ (Fig. 4c). In addition, the PIF-RS gene set is enriched in *GLK1*-regulated genes, and *GLK1*-bound genes are enriched in PIF-RS genes (Supplementary Fig. 17). Taken altogether, these results indicate that *GLK1* is transcriptionally targeted, in antagonist fashion, by informational-light and GUN1-facilitated-retrograde signals, which induce and repress *GLK1* expression, respectively, to regulate a subset of PIF-RS-controlled genes. Our results also indicate that *GLK1* is not regulated under these conditions by either ABI4 (Supplementary Fig. 18; in agreement with our findings presented in Fig. 3c,d) or by HY5, which was previously proposed to respond to RS^13^ (Supplementary Fig. 19).

To test whether the transcriptional regulation of *GLK1* by light and RS might be relevant to seedling photomorphogenesis, we examined the phenotype of seedlings with altered *GLK1* expression. We reasoned that if GLK1 is a key link regulating photomorphogenesis downstream of both sensory-photoreceptor-mediated light signalling and RS, *glk1* mutants should exhibit alterations in response to activation of these pathways. In agreement with this proposal, *glk1* seedlings were indistinguishable from the WT in the dark but displayed longer hypocotyls and less separated cotyledons when grown in the light (Fig. 4d; Supplementary Fig. 20), indicating that GLK1 acts positively in seedling photomorphogenic development. Seedlings constitutively expressing *GLK1* under the control of the 35 S promoter (*GLK1-OX*), were similar to WT in the dark although did show slightly shorter hypocotyls (Supplementary Fig. 20b), but, in striking contrast to WT, partially deetiolated in the presence of lincomycin in response to light, as indicated by short hypocotyls, and open and expanded cotyledons (Fig. 4e,f), and displayed high *LHCB* expression in lincomycin-treated seedlings, similarly to *gun1* (Supplementary Fig. 21). In contrast to *GLK1*, the closely related *GLK2* gene appears to have a minor role in this process (Supplementary Fig. 22). These findings extend and refine the previously described role of GLK1 as a regulator of photosynthetic-apparatus-gene expression^17^, to define it as a previously unrecognized positive factor that acts pleiotropically to orchestrate the broader photomorphogenic programme. Altogether with the evidence that *GLK1* is a direct target of PIF-imposed repression, the data support the conclusion that *GLK1* is a pivotal target directly at the convergence of the informational-light/PIF and RS/GUN1 signalling pathways. This conclusion implies strongly in turn, that the GUN1-facilitated, RS-imposed repression of the light-induced expression of *GLK1*, that is otherwise necessary for deetiolation, provides a mechanism to attenuate seedling photomorphogenesis in the event of chloroplast disruption. Such disruption has been shown to activate RS under natural environments triggered by excess light^33^,^34^, highlighting the likely biological significance of this proposed attenuation mechanism.

### High-intensity light inhibits PIF-mediated deetiolation

**Figure 5:**
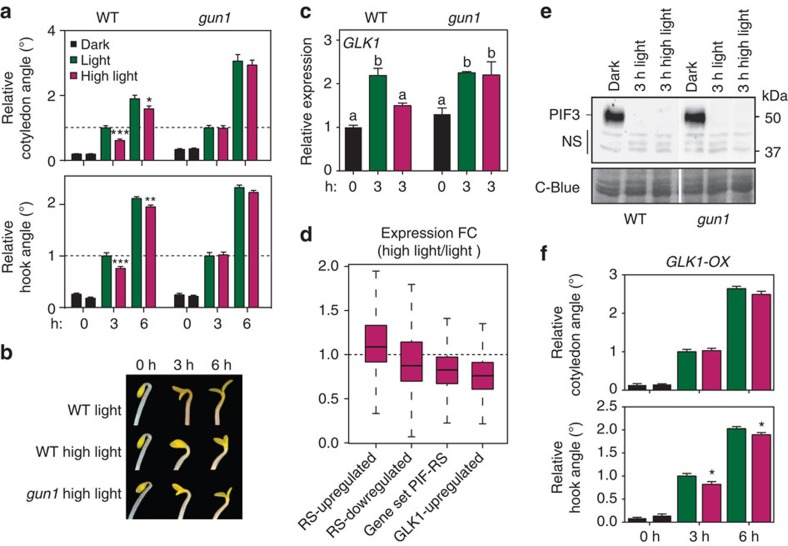
High-intensity light induces GUN1-facilitated RS and inhibits PIF-mediated early light-induced deetiolation. (**a**) Hook unfolding and cotyledon separation were significantly slower in dark-grown WT seedlings after 3 and 6 h of high light treatment (purple) compared with light (green), while *gun1* mutant seedlings were insensitive to high light and deetiolated at the same rate in both light and high light. Values for each genotype are expressed relative to the corresponding value in light at 3 h set at one (*n*⩾65). (**b**) Visual phenotype of representative seedlings in **a**. (**c**) Light-induction of *GLK1* in dark-grown WT seedlings is significantly reduced in high light (purple) compared with light (green), while *gun1* mutant seedlings are insensitive to high light and reached similar levels of *GLK1* induction in light and high light. Data are the means±s.e.m. of biological triplicates (*n*=3). Different letters denote the statistically significant differences among means (Tukey-b test). (**d**) Fold-change expression between WT seedlings grown in high light and light for the following gene sets: RS-upregulated^27^, RS-downregulated^27^, Gene-Set PIF-RS and GLK1-upregulated^17^. Data were obtained from ref. 35. (**e**) PIF3 protein levels in WT and *gun1* dark-grown seedlings were below level of detection after 3 h in light or in high light. (**f**) *GLK1-OX* seedlings were largely insensitive to high light and deetiolated similarly in both light (green) and high light (purple; *n*⩾45). In **a** and **f**, error bars represent s.e.m. and the statistically significant differences between light treatments at each time point by Student's *t*-test are shown (*P<0.05; **P<0.01 and ***P<0.001). The experiments were repeated two times with similar results. C-blue, coomassie blue; NS, non-specific bands.

To investigate the proposed mechanism and the role of PIF-RS-regulated *GLK1* expression more closely, we examined the consequences of exposing dark-grown seedlings to RS-inducing high light intensity (high light, 310 μmols m^−2^ s^−1^; Supplementary Fig. 23), compared with lower light levels (130 μmols m^−2^ s^−1^, defined here as ‘low light' for convenience). Under high light intensity, we detected GUN1-mediated repression of deetiolation within 3 h (Fig. 5a,b), consistent with the effect of lincomycin on early deetiolation responses to the dark-to-low light transition (Fig. 1c). Under these conditions, we observed that the rapid, low light induction of *GLK1* expression was significantly reduced by high light intensity, whereas induction levels of *GLK1* expression in *gun1* were similar in low and high light (Fig. 5c, Supplementary Fig. 24). This result suggests that high light intensities can antagonize the low-light induction of *GLK1* expression, at least in part through GUN1-mediated RS. In agreement with this conclusion, analysis of the available data^35^ indicates that high light intensities prevent the informational low-light induction of GLK1-induced genes, a behaviour that is similar for the genes in ‘Gene-Set PIF-RS' or the genes whose light-induction is inhibited by RS (Fig. 5d). Because PIF3 levels in the WT and *gun1* were below the level of detection in both low and high light (Fig. 5e), and *pifq* deetiolation and GLK1 induction was slower under high light than low light (Supplementary Fig. 25), our findings collectively support the notion that *GLK1* expression under high light is repressed in a PIF-independent fashion by a GUN1-facilitated pathway, to antagonize the light induction of the photomorphogenic programme. Moreover, in agreement with the role we propose for GLK1 as downstream effector of both informational-light and RS, we found that *GLK1-OX* seedlings were largely insensitive to high light intensity and underwent deetiolation similarly in both low and high light (Fig. 5f). On the basis of these assembled data, we conclude that activation of GUN1-mediated RS by high light during early deetiolation represses informational-light-induced derepression of *GLK1*, attenuating hook unfolding and cotyledon separation. This might protect the seedling by minimizing the exposed cotyledon surface to avoid excess light damage, adding to other high light-induced strategies like excess light dissipation^33^,^36^. Consistent with this notion, survival of *gun1* mutants during deetiolation under high light is significantly poorer than WT^13^,^37^.

## Discussion

**Figure 6:**
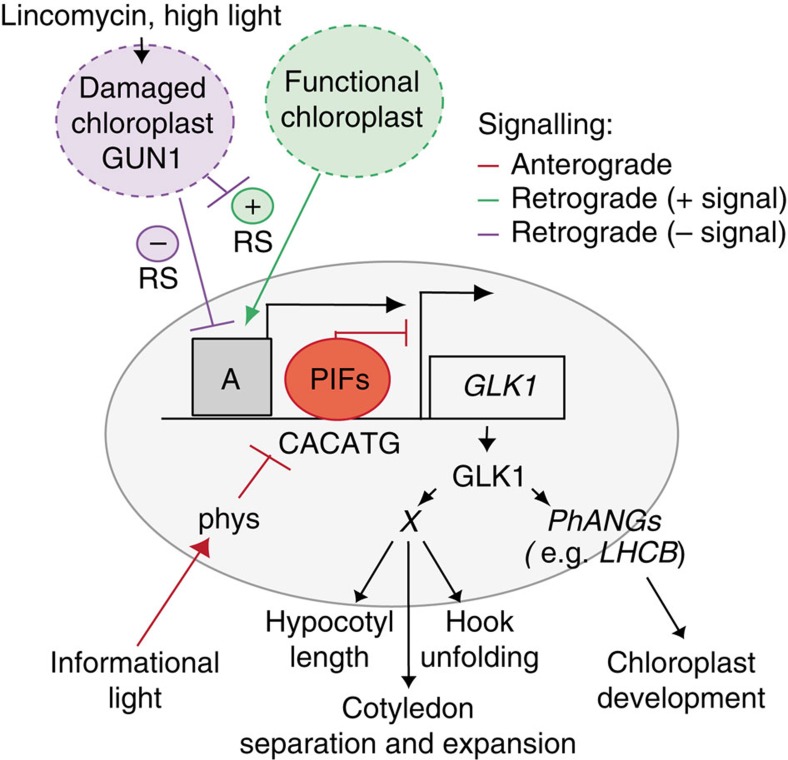
Antagonistic actions of PIF-mediated light signalling and GUN1-mediated plastid RS in regulating photomorphogenesis. PIFs bind to the *GLK1* promoter through a PBE motif (CACATG)^16^ to directly repress *GLK1* expression in the dark. Unknown transcriptional activator(s) represented by A sits on the promoter constitutively poised to activate *GLK1* expression. In response to the informational light signals, activated phytochromes induce degradation of the PIFs, triggering the derepression of *GLK1* expression, driven by A. In turn, *GLK1* directly induces expression of photosynthesis-associated nuclear genes (*PhANGs*)^17^, and of one or more putative ‘*Gene(s) X*' that implement other aspects of photomorphogenesis. If chloroplast integrity is disrupted by lincomycin or high light, a negative retrograde signal ((−) RS) emitted by dysfunctional chloroplasts induces GUN1-mediated repression of *GLK1* expression by repressing the effectiveness of A. Alternatively, functionally intact chloroplasts might produce a positive RS ((+) RS) necessary for the expression and/or activity of A, that is disrupted in a GUN1-facilitated manner when chloroplast function is altered.

Our findings support a mechanistic model (Fig. 6), whereby the PIFs directly repress *GLK1* expression in the dark to support skotomorphogenesis. In the light, phytochrome-induced degradation of the PIFs relieves the repression of *GLK1* expression and this permits initiation of photomorphogenic development, as long as the plastid is functionally intact, through GLK1 regulation of photosynthetic genes^17^ and potentially of as yet undefined genes involved in the regulation of other facets of photomorphogenesis (putative *‘Gene(s) X'* in Fig. 6). In conditions where the plastid is damaged, RS is activated and antagonizes the phytochrome-signal output by repressing the light-induced derepression of *GLK1*, through a GUN1-facilitated, PIF-independent pathway, which effectively attenuates normal photomorphogenesis for the purpose of protecting the seedling. According to this model, whereas the phytochrome/PIF system monitors dark-light transitions, as well as light quality and periodicity, to optimize light-regulated development, the chloroplast functions as a sensor of excess light (at levels where the phytochrome/PIF system is saturated), to prevent potentially irreversible damage. Hence, coincidence of external light stimuli and internal chloroplast integrity is necessary to promote photomorphogenesis.

Activation of RS has been proposed, alternately, to involve both positively and negatively acting configurations^30^,^38^. In principle, our data are consistent with either possibility, in which lincomycin would, respectively, either (a) disrupt a positive intact-plastid-emitted signal, which acts in a GUN1-regulated manner and is necessary for the expression of *GKL1* following derepression by PIF removal, or (b) induce a negative plastid-emitted signal, which acts to repress the nuclear transcription of *GLK1* in a GUN1-mediated manner (Fig. 6). In either configuration, our observation that the *cop*-like phenotype of *pifq* is suppressed by lincomycin in the dark suggests that activation of RS by plastid malfunction is independent of light, consistent with previous reports of RS activity in darkness^19^,^27^. Although the mechanism by which RS repress light-induced derepression of *GLK1* expression remains unknown, our results establish that the process requires GUN1, is independent of the PIFs, and does not appear to involve GUN5, GUN6 or ABI4 (in agreement with Kakizaki *et al*.^18^), and might involve regulation at the transcriptional and/or post-transcriptional levels through a factor or factors (‘A' in our model in Fig. 6) of yet unknown nature.

## Methods

### Plant materials and growth conditions

*Arabidopsis thaliana* seeds used in these studies have been described elsewhere, including *pifq* (ref. 10), *glk1.1* (ref. 39)*, GLK1-OX and GLK2-OX* lines (ref. 40), *abi4-t* (ref. 41), *gun5-1* (ref. 21), *gun6-1D* (ref. 30), *cop1-4* (ref. 42) and *hy5-215* (ref. 43), all in the Columbia (Col-0) ecotype, and *sigma2-1* (ref. 44) and *sigma6 (soldat8*; ref. 45) in the WS and L*er* and ecotypes, respectively. The newly described *gun1* allele (*gun1-201*) corresponds to insertion line SAIL_290_D09 obtained from ABRC (Supplementary Fig. 26). Seeds were sterilized and plated on medium without sucrose as described^46^. Seedlings were then stratified for 4 days at 4 °C in darkness, and then placed under continuous white light (1 μmol m^−2^ s^−1^), red light (1.3 μmol m^−2^ s^−1^) or darkness for 3 days, except in experiments shown in Fig. 1c, performed using a white light intensity of 25 μmol m^−2^ s^−1^, and in Fig. 5, performed using a combination of red (60%) and blue (40%) light, where Light corresponds to 130 μmol m^−2^ s^−1^ and High light to 310 μmol m^−2^ s^−1^. Fluence rates were measured with a Spectrosense2 metre associated with a 4-channel sensor^47^. For lincomycin treatments, media was supplemented with 0.5 mM lincomycin (Sigma L6004) or as indicated (Supplementary Fig. 4)^19^. For norflurazon treatment, media was supplemented with 5 uM norflurazon (Novartis 100–848-AA). To measure hypocotyl length, hook angle and cotyledon angle and area, seedlings were arranged horizontally on a plate and photographed using a digital camera (Nikon D80). Measurements were performed using NIH Image software (Image J, National Institutes of Health)^48^. At least 25 seedlings were measured to calculate the mean and s.e.m. in at least two biological replicates. In Supplementary Fig. 25, the response to light was measured in individual *pifq* seedlings across time.

### Gene expression analysis

Quantitative RT–PCR, RNA extraction, cDNA synthesis and qRT–PCR were done as described^48^. Briefly, 1 μg of total RNA extracted using the RNeasy Plant Mini Kit (Qiagen) were treated with DNase I (Ambion) according to the manufacturer's instructions. First-strand cDNA synthesis was performed using the SuperScript III reverse transcriptase (Invitrogen) and oligo dT as a primer (dT30). cDNA was then treated with RNase Out (Invitrogen) before 1:20 dilution with water, and 2 μl was used for real-time PCR (Light Cycler 480; Roche) using SYBR Premix Ex Taq (Takara) and primers at a 300 nM concentration. Gene expression was measured in three independent biological replicates (with the exception of Supplementary Fig. 6b (one), Supplementary Fig. 23 (four) and Supplementary Figs 24 and 25 (two)), in at least three technical replicates for each biological sample. *PP2A* (*AT1G13320*) was used for normalization^49^. Primers used to analyse *LHCB1.4* (*AT2G34430*) and *LHCB2.2* (*AT2G05070*) were described previously^10^,^27^. *GLK1* (*AT2G20570*) expression was measured using primers 5′- GCTACGAGATTTAGAGCACCG -3′ and 5′- TTGACGGATGTAAGTCTACCG -3′, and *GUN1* (*AT2G31400*) expression using primers 5′- TGAGTATATTGACTGGCTGGG -3′ and 5′- GCATTTTGACAGGTGGAATGG -3′.

### RNA-seq library construction and data processing

Total RNA from 3-day-old dark-grown seedlings was extracted using QIAshredder columns and the RNeasy Plus Mini Kit (Qiagen). RNA-seq libraries were prepared using Illumina's directional mRNA-seq sample preparation following the manufacturer's protocol with some modifications. The mRNA was purified from 20 μg of total RNA using Dynabeads Oligo (dT)_25_ (Invitrogen) and fragmented using Fragmentation Reagents (Ambion). The resulting polyA-tailed 3′-end fragments were captured using Dynabeads Oligo (dT)_25_ (Invitrogen), and then treated by Antarctic Phosphatase (NEB) and T4 Polynucleotide Kinase (NEB). The sample was purified using RNeasy MinElute Cleanup Kit (Qiagen) and Illumina's SRA 5′-adaptor was ligated to the eluted mRNA fragments by T4 RNA Ligase 1 (NEB). Reverse transcription was performed using the SuperScript III First-Strand Synthesis System (Invitrogen) and the 3′-cDNA adaptor derived from Illumina's v1.5 sRNA 3′-adaptor conjugated with the anchored oligo (dT)_20_ primer. The first-strand cDNA was purified using the Agencourt AMPure XP system. The second-strand cDNA was synthesized and amplified by PCR using Phusion High-Fidelity DNA Polymerase with Illumina's sRNA PCR primer set. The library was purified using the Agencourt AMPure XP system and the size was validated by Bioanalyzer 2000 (ref. 50). Libraries from triplicate biological samples were sequenced on the Illumina HiSeq platform. Reads were aligned to the TAIR10 representative transcriptome using Bowtie^51^ with one mismatch allowed. To prevent false counts, mapping was performed using the 3′-end 500-bp region of the coding strand. Differentially expressed genes were identified using the edgeR package^52^ among those genes in which at least 2 of the 6 samples being compared had⩾5 reads per million. SS genes were defined as those that differ with a *P* value≤0.05 (adjusted for false-discovery rate), and SSTF genes as those that differ by more than or equal to twofold with a *P* value≤0.01 (adjusted for false-discovery rate)^11^ (Supplementary Fig. 27).

### Microarray data comparison and transcript analysis

Expression data shown in Figs 2b, 3c,d and 4c, and Supplementary Figs 8b, 11b and 18b, were obtained from microarray data from GSE5770 (ref. 23) and GSE17159 (ref. 11). As a control, gene expression in the shared WT Light sample from the two experiments were first compared in each subgroup of genes analysed in each figure to validate that there were no statistically significant differences and thus the rest of the samples were comparable. Represented WT light values in Figs 2b and 4c, and Supplementary Figs 8b, 11b and 18b, are from ref. 23. Expression data shown in Fig. 5d were obtained from GSE7743 (ref. 35).

### Promoter analysis for DNA-binding motifs

Analysis was performed using the ‘Motif Analysis' tool available at The Arabidopsis Information Resource (http://Arabidopsis.org/tools/bulk/motiffinder/index.jsp) for statistically overrepresented 6-mer motifs in the 500-bp genomic sequence upstream of the start codon of genes in gene-set PIF-RS, the SCOPE motif finder (http://genie.dartmouth.edu/scope/), and the *Arabidopsis* Gene Regulatory Information Server ‘Agris' (http://arabidopsis.med.ohio-state.edu/AtTFDB/).

### Statistics

Gene expression and morphological data shown in Figs 1b, 4 and 5c, and Supplementary Figs 2, 5b, 6c, 7, 14b, 15b, 18a, 19a, 22b, 24 and 25b, were analysed by one-way analysis of variance, and the differences between means were evaluated using Tukey-b *post hoc* multiple comparison test (IBM SPSS Statistics Software). Statistically significant differences were defined as those with a *P* value<0.05. Morphological and expression data were analysed using Excel (Microsoft) for statistically significant differences from their control. *P* values were determined by homoscedastic Student's *t*-test for data in Figs 1c, 4b,d and 5a,f, and Supplementary Figs 1b, 4, 6b, 20, 21, 22c, 25a and 26b, and WT and *pifq* in Fig. 1e, and by heteroscedastic Student's *t*-test for data in Figs 2b, 3c and 4c, and Supplementary Figs 8b and 18b. Statistically significant differences were defined as those with a *P* value<0.05. In the figures significance level is indicated as *P<0.05, **P<0.01 and ***P<0.001. Hypergeometric tests shown in Fig. 4a and Supplementary Fig. 17 were performed using R.

### Protein extraction and immunoblots

Protein extracts were prepared from 2-day-old dark-grown WT and *gun1* seedlings transferred to white light for the time and light intensities indicated in Fig. 5e. Tissue samples were collected and frozen in liquid nitrogen, and samples were manually ground under frozen conditions before resuspension in extraction buffer. Protein extraction was performed in boiling extraction buffer (100 mM MOPS (pH 7.6), 2% SDS, 10% glycerol, 4 mM EDTA, 2 g l^−1^ aprotinin, 3 g l^−1^ leupeptin, 1 g l^−1^ pepstatin and 2 mM PMSF). Total protein was quantified using a Protein DC kit (Bio-Rad), and β-mercaptoethanol was added just before loading^53^. Aliquots from each sample containing equal amounts of total protein (150 μg) were subjected to 7.5% SDS-PAGE gels. Proteins were then transferred to Immobilon-P membrane (Millipore), and immunodetection of endogenous PIF3 was performed as previously detailed^54^ using a rabbit anti-PIF3 polyclonal antibody^55^ (1:10,000 dilution) incubated with Hikari solution (Nacalai Tesque). Peroxidase-linked anti rabbit secondary antibody (1:5,000 dilution; Amersham Biosciences NA934) and a SuperSignal West Femto chemiluminescence kit (Pierce) were used for detection of luminescence using LAS-4000 Image imaging system (Fujifilm). The membrane was stained with Coomassie blue as a loading control. The uncropped scan of the western blot shown in Fig. 5e is provided in Supplementary Fig. 28.

### Transmission electron microscopy

Cotyledons from 3-day-old dark-grown seedlings were fixed and processed as described in ref. 11, except that a Leica EM PACT2-RTS high-pressure freezing machine (Leica Microsystems, Vienna, Austria) was used for the high-pressure freezing method. Ultrathin sections were visualized in a Jeol JEM1010 electron microscope (JEOL Ltd, Akishima, Tokyo, Japan). Images were recorded with a SIS Mega View III CCD camera.

## Additional information

**Accession codes:** RNA-seq data reported in this study have been deposited in the Gene Expression Omnibus database under the accession number GSE78969.

**How to cite this article:** Martín, G. *et al*. Phytochrome and retrograde signalling pathways converge to antagonistically regulate a light-induced transcriptional network. *Nat. Commun.* 7:11431 doi: 10.1038/ncomms11431 (2016).

## Supplementary Material

Supplementary Figures and Note Supplementary Figures 1-28 and Supplementary Note 1

Supplementary Data Set 1 Analysis and expression data of genes co-regulated by Retrograde Signaling, Light treatment and PIF transcription factors. Co-regulation is reported in Fig. 2a and Supplementary Fig. 8a. PIF and Light regulated genes were defined previously in ^11^. Retrograde Signaling regulated genes were previously reported in ^27^. Expression data was obtained from GSE17159 and GSE5770 respectively and are shown in Figs 2b, 3c,d and 4c, and Supplementary Figs 8b, 11b, and 18b. PIF bound genes were defined previously in ^16^. Subcellular localization is obtained from subcellular predictions available in TAIR (http://www.Arabidopsis.org).

Supplementary Data Set 2 PIF and Lincomycin regulated genes in 3 day-old dark-grown seedlings. List of SSTF genes defined as those that differ by ≥2-fold with P values (adjusted for false discovery rate) ≤0.05 in WT dark vs pifq dark, and SS genes, set as those expressed statistically significantly different with P values (adjusted for false discovery rate) ≤0.05, comparing *pifq* dark VS *pifq* dark Lincomycin. Expression data are shown in Fig. 2c, and Supplementary Figs 9, 10, and 11b. PIF bound and Retrograde Signaling (RS) regulated genes were defined previously in ^16^ and ^27^ respectively. Subcellular localization is obtained from subcellular predictions available in TAIR (http://www.Arabidopsis.org)

Supplementary Data Set 3 Lincomycin-regulated genes in 3 day-old dark-grown WT and *pifq* seedlings. List of SSTF genes defined as those that differ by ≥2-fold with P values ≤0.01 (adjusted for false discovery rate, FDR) in WT dark vs WT dark lincomycin or *pifq* dark vs *pifq* dark lincomycin. This analysis is reported in Supplementary Fig. 27. Retrograde signaling (RS)-regulated genes were defined previously in ^27^.
