# Supplementary material for: Phytochrome and retrograde signalling pathways converge to antagonistically regulate a light-induced transcriptional network
Source: Nat Commun. 2016 May 6;7:11431. doi: 10.1038/ncomms11431 (PMC4859062; doi:10.1038/ncomms11431)
Supplement: Supplementary Figures and Note — Supplementary Figures 1-28 and Supplementary Note 1 [file ncomms11431-s1.pdf]

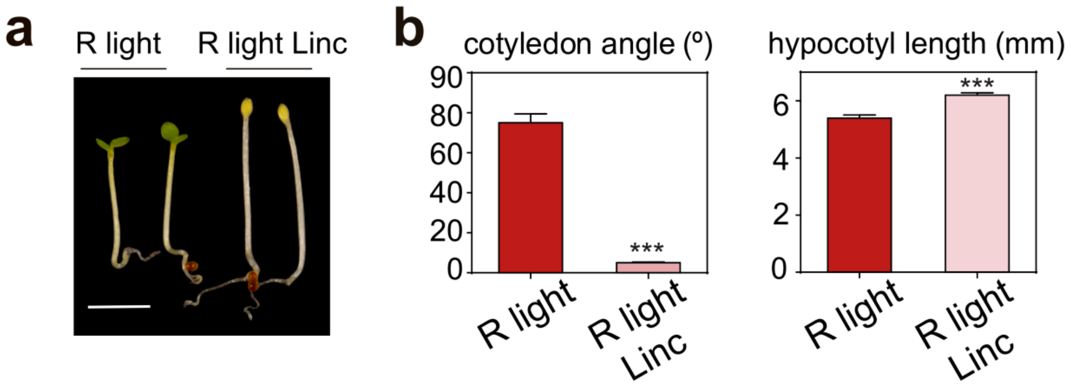

Supplementary Fig. 1. Retrograde signals (RS) from the chloroplast suppress seedling deetiolation under continuous Red light. (**a**) Lincomycin treatment prevents Arabidopsis seedling deetiolation in continuous red (R) light. Wild-type seedlings were grown for 3 days in the light in the absence (R Light) or presence (R Light Linc) of lincomcyin. Scale bar corresponds to 2.5 mm. (**b**) Cotyledon angle and hypocotyl length of seedlings grown as in (a). Error bars represent SE of two independent experiments, each with n≥30. Asterisks indicate statistically significant differences from red light-grown WT seedlings by Student's *t* test ($P < 0.001$ (***)).

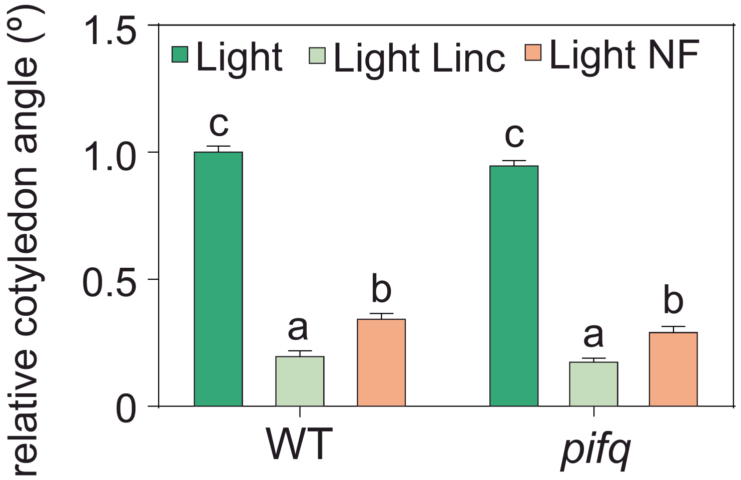

Supplementary Fig. 2. Norflurazon treatment suppresses seedling deetiolation under light conditions. Cotyledon angle of wild-type (WT) and *pifq* seedlings grown for 3 days in the light in the absence (Light) and presence of either lincomcyin (Light Linc) or norflurazon (Light NF). Values were normalized to the WT light-grown seedlings. Error bars represent SE of 25 seedlings. The experiment was repeated two times with similar results. Different letters denote statistically significant differences among means by Tukey-b's test ($P<0.05$).

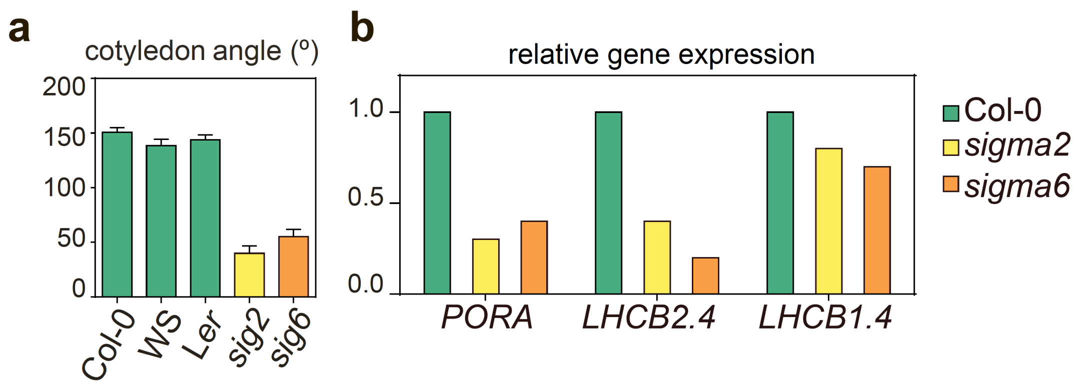

Supplementary Fig. 3. Mutations in *SIGMA2* and *SIGMA6*, known to activate chloroplast retrograde signaling, suppress seedling deetiolation in light. (**a**) Cotyledon angle of wild-type Col-0, WS, and L*er* seedlings, and *SIGMA2* (*sig2*) and *SIGMA6* (*sig6*) mutant seedlings grown for 3 days in white light. Error bars represent SE of two independent experiments, each with n≥20. The background ecotypes for *sig2* and *sig6* mutants are WS and L*er* respectively (see Methods). (**b**) Expression of representative plastid retrograde signaling marker genes *PORA*, *LHCB2.4* and *LHCB1.4* in light-grown Col-0, *sig2* and *sig6* seedlings relative to the Col-0 value set as one. Data are from [27].

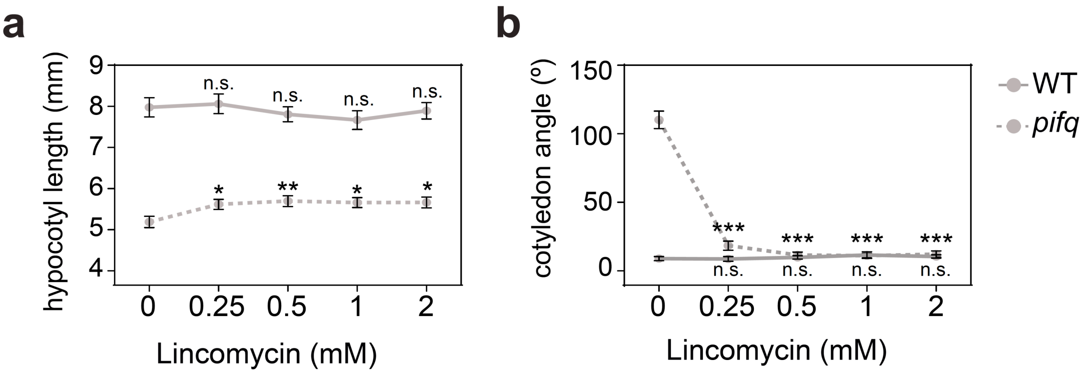

Supplementary Fig. 4. Dose-dependent reversion of the constitutive photomorphogenic phenotype of dark-grown *pifq* seedlings by lincomycin. Hypocotyl length (**a**) and cotyledon angle (**b**) of 3-day-old dark-grown WT and *pifq* seedlings in the presence of increasing lincomycin concentrations as indicated. Error bars represent SE, (n at each point ≥30). Asterisks indicate statistically significant differences in each genotype from the 0 mM lincomycin point by Student's *t* test (P < 0.05 (*); P < 0.01 (**); P < 0.001 (***); n.s., not significant).

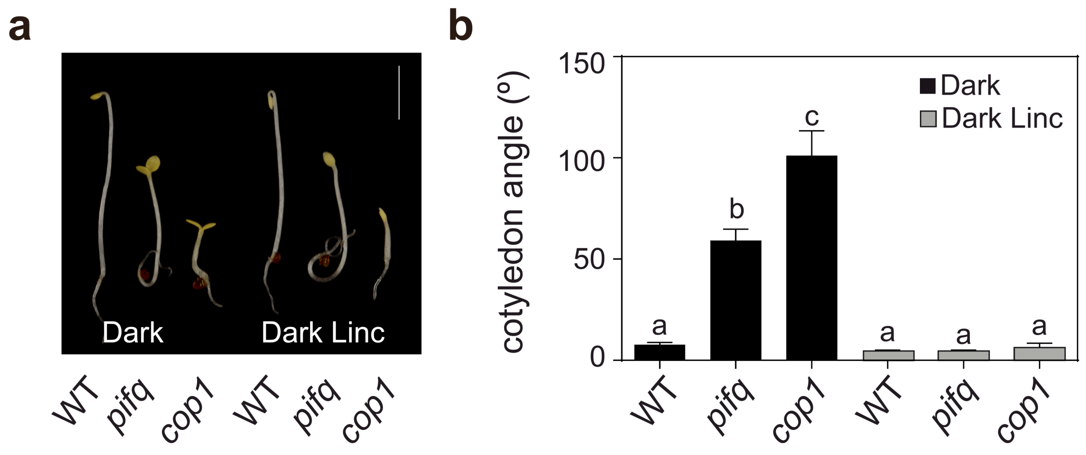

Supplementary Fig. 5. Retrograde signals from the chloroplast suppress the constitutive photomorphogenic phenotype of *pifq* and *cop1* seedlings in the dark. **(a)** Wild-type, *pifq* and *cop1-4* seedlings were grown for 3 days in the dark in the absence (Dark) or presence (Dark Linc) of lincomcyin. Scale bar corresponds to 2.5 mm (left). (**b**) Cotyledon angle of seedlings grown as in (a). Error bars represent SE (n≥30, except for *cop1* where n=7). The experiment was repeated three times with similar results. Different letters denote statistically significant differences among means by Tukey-b's test (P <0.05).

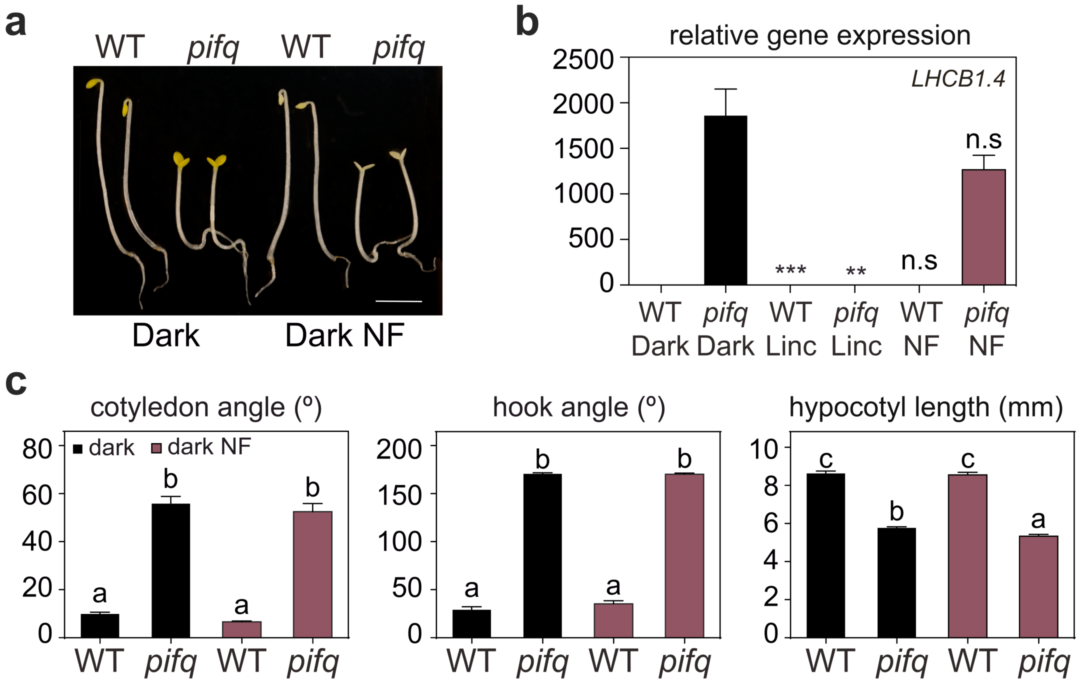

Supplementary Fig. 6. Norflurazon treatment does not affect the development of dark-grown seedlings. (**a**) Wild-type and *pifq* seedlings were grown for 3 days in the dark in the absence (Dark) or presence (Dark NF) of norflurazon. (**b**) Expression of *LHCB1.4* was analyzed by qRT-PCR in 3-day-old WT or *pifq* seedlings grown for 3 days in the dark in the absence (Dark) and presence of either lincomcyin (Linc) or norflurazon (NF). Values were normalized to *PP2A*, and expression levels are expressed relative to WT dark set at one. Data are the means ± SE of technical triplicates. n.s indicate not statistically significant differences of norflurazon treatment in each genotype by Student's *t* test (P < 0.01 (**); P < 0.001 (***); n.s., not significant). (**c**) Cotyledon and hook angle, and hypocotyl length of seedlings grown as in (a). Error bars represent SE of two independent experiments (n≥25). Different letters denote statistically significant differences among means by Tukey-b's test (P <0.05).

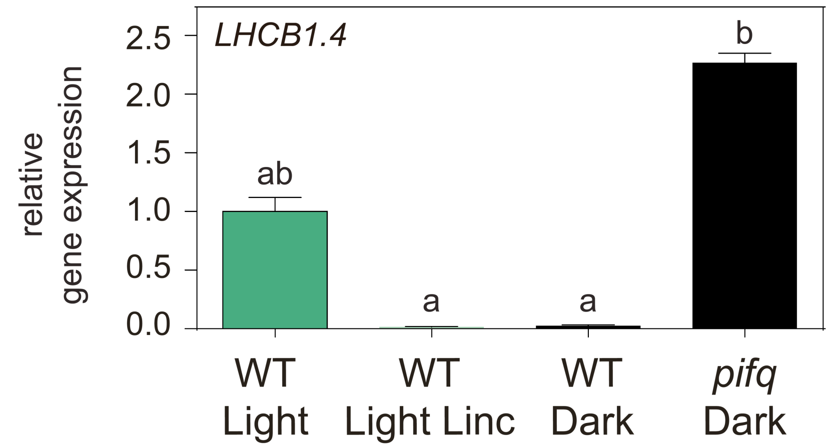

Supplementary Fig. 7. Lincomycin treatment represses induction of *LHCB1.4* in continuous white light to levels similar to dark. Expression of *LHCB1.4* was analyzed by qRT-PCR in 3-day-old WT or *pifq* seedlings grown in the absence of lincomycinin the dark (Dark), in continuous white light (Light), or in the presence of lincomycin in continuous white light (Light Linc). Values were normalized to *PP2A*, and expression levels are expressed relative to WT light set at one. Data are the means ± SE of biological triplicates. Different letters denote statistically significant differences among means by Tukey-b's test ($P <0.05$).

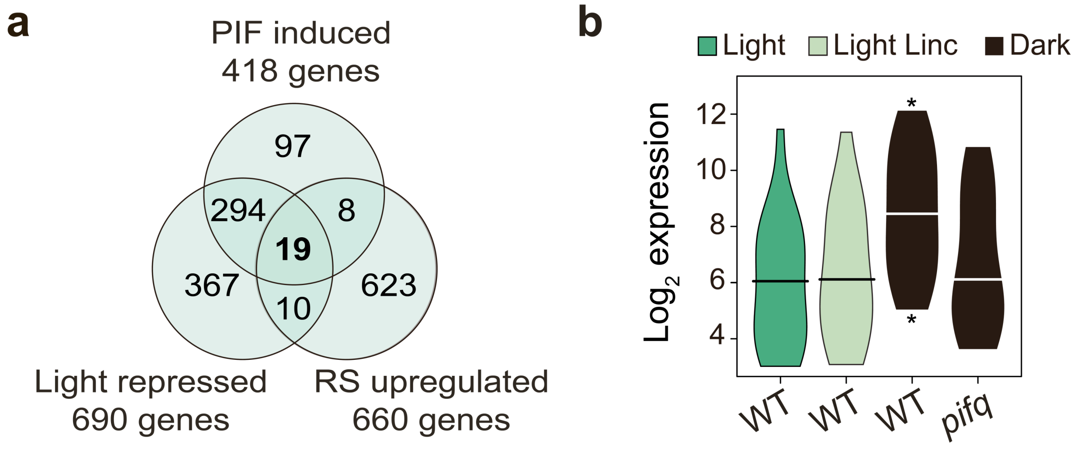

Supplementary Fig. 8. Retrograde signaling is a minor regulator of the expression of light-repressed PIF-induced genes. (**a**) Venn diagram showing poor overlap among RS-upregulated [27], light-repressed [11], and PIF-induced [11] genes. The list of genes that belongs to each subgroup is provided in Supplementary Data 1. (**b**) Vioplot representation of mRNA levels of the 19 overlapping genes shown in (a) in 2 day-old dark-grown WT and *pifq* seedlings (in black), and 5 day-old light-grown WT grown in absence (dark green) or presence (light green) of lincomycin. Statistically significant differences by heteroscedastic *t* test from light-grown WT in the absence or presence of lincomycin are indicated in the upper and lower part respectively ($P < 0.05$ (*)). Data were obtained from [11, 23].

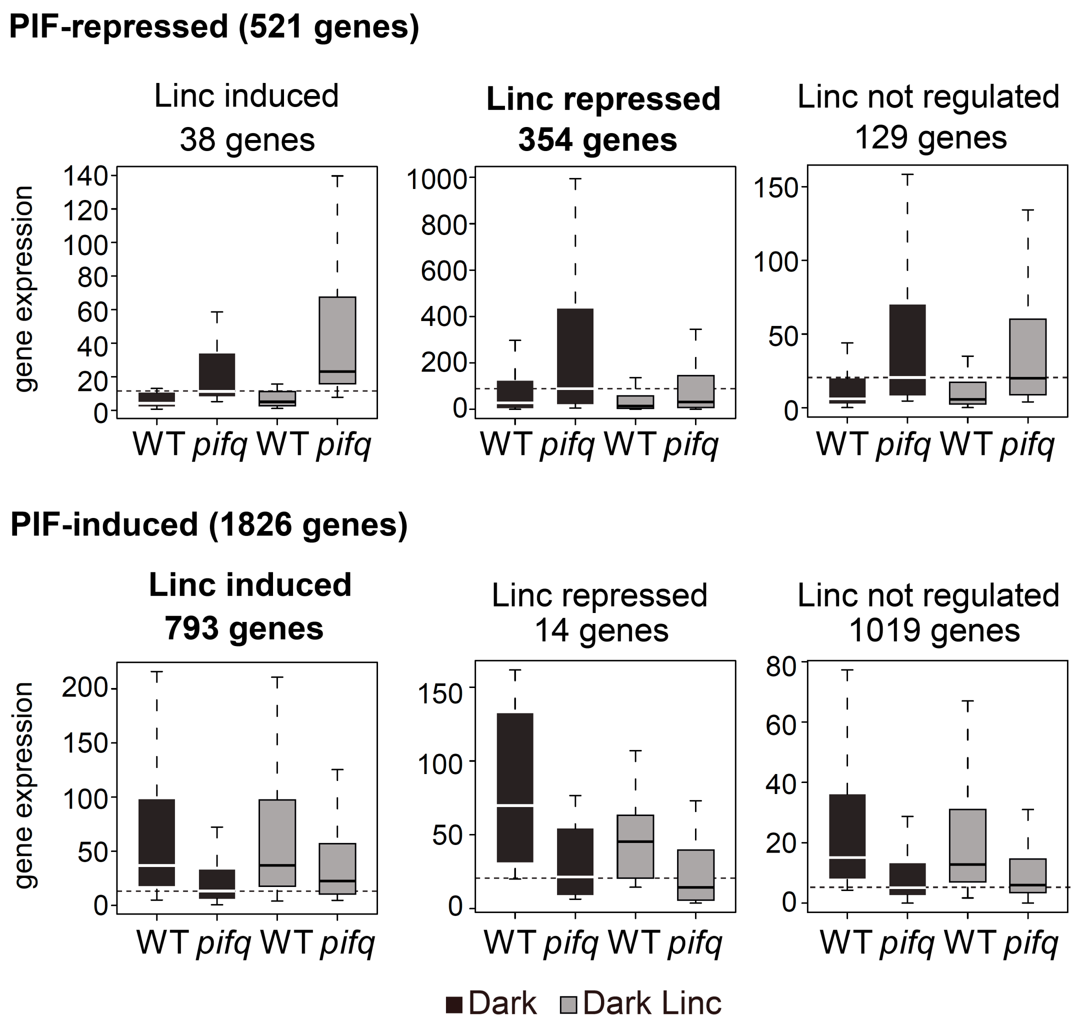

Supplementary Fig. 9. Lincomycin and PIFs regulate gene expression in the same direction. Transcript levels of 521 SSTF PIF-repressed (top) and 1826 SSTF PIF-induced (bottom) genes in 3 day-old dark-grown WT and *pifq* seedlings in the absence (Dark) (black boxes) or presence (Dark Linc) (gray boxes) of lincomycin. Groups are based on their response to lincomycin: statistically and significantly (SS) induced (left), repressed (center), or not SS regulated by lincomycin (right). *pifq* median in the dark is represented with a black dashed line. The list of genes belonging to each subgroup is provided in Supplementary Data 2. SSTF genes: genes whose expression changed statistically significantly and by at least twofold.

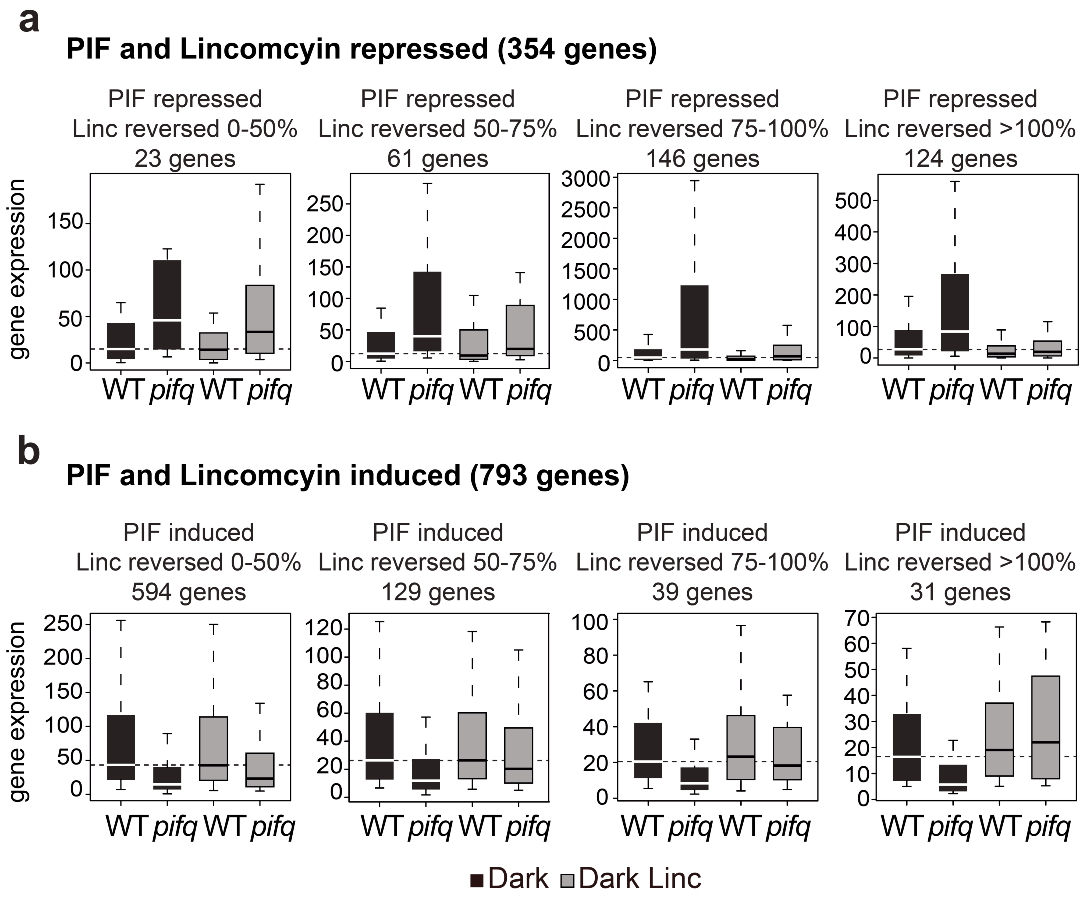

Supplementary Fig. 10. Lincomcyin reversion of PIF-regulated gene expression. Transcript levels of genes regulated in the same direction by PIFs (SSTF genes) and lincomycin (SS genes) (described in Supplementary Fig. 9) in 3 day-old dark-grown WT and *pifq* seedlings in the absence (Dark) (black boxes) or presence (Dark Linc) (gray boxes) of lincomycin grouped by the percentage of reversion to WT levels induced by lincomycin: less than 50% (left), between 50 and 75% (middle left), between 75 and 100% (middle right) or more than 100% (right). (**a**) 354 PIF- and lincomycin-repressed genes. (**b**) 793 PIF- and lincomycin-induced genes. WT median in the dark is represented by a black dashed line. The list of genes belonging to each subgroup is provided in Supplementary Data 2. SS and SSTF genes: genes whose expression changed statistically significantly (SS) and by at least twofold (SSTF).

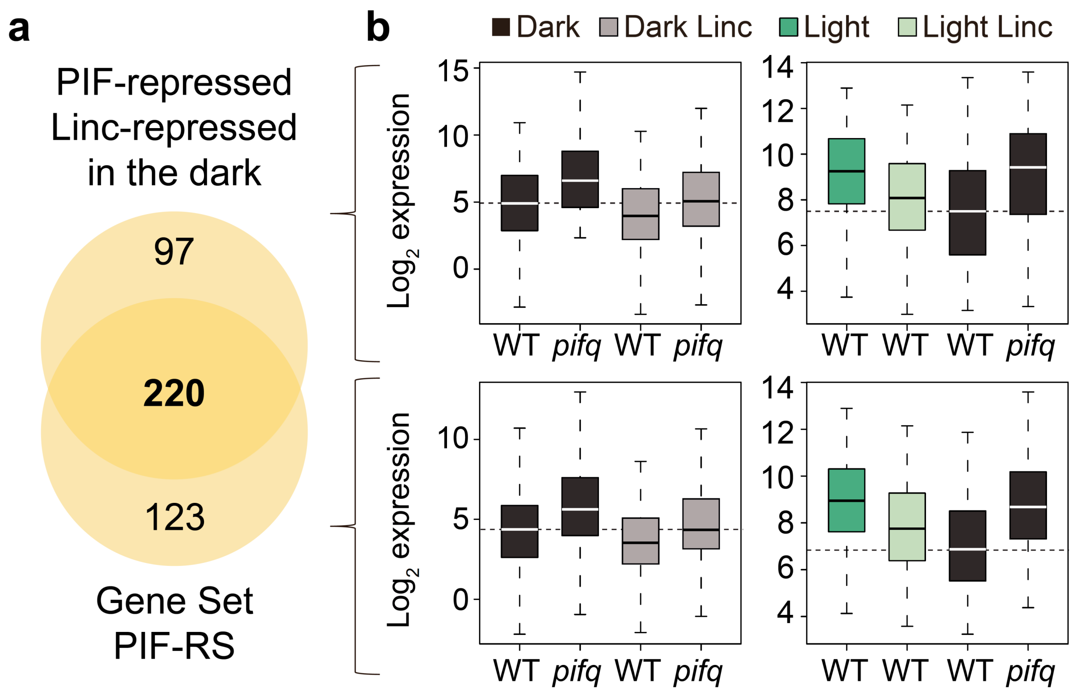

Supplementary Fig. 11. PIF- and lincomycin-repressed genes in the dark extensively overlap with genes in the PIF-RS gene set. (**a**) Venn diagram showing overlap between the 354 PIF- and lincomycin (linc)-repressed gene set (Fig. 2c) in the dark and the 343 genes defined as 'gene set PIF-RS' (Fig. 2a). Only 317 out of 354 are present in the ATH1 array and were used here for the comparison. (**b**) Boxplot representation of the transcript levels of the 354 PIF- and lincomycin-repressed genes in darkness (top) and the 343 genes in gene set PIF-RS (bottom), in 3 day-old dark-grown wild type (WT) and *pifq* grown in the absence (dark) or presence (grey) of lincomycin (Linc) (left), and in 5 day-old light-grown WT in absence (dark green) or presence (light green) of lincomycin compared to 2 day-old dark-grown WT and *pifq* (black) (right). The median value in WT dark is represented by a black dashed line. Available data for the 343 PIF-RS geneset was obtained from [11, 23].

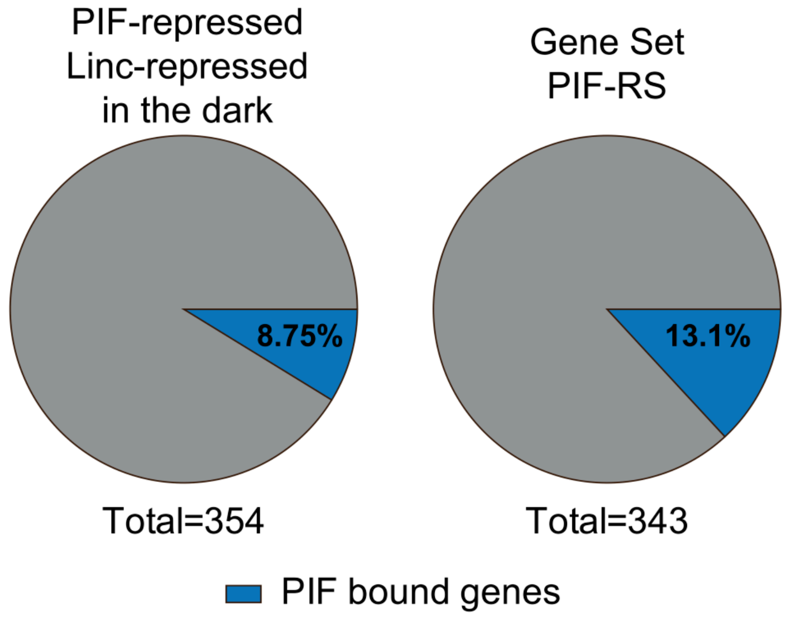

Supplementary Fig. 12. Percentage of PIF-bound genes in PIF- and RS-repressed genes. Percentage of PIF-bound genes in the light/PIF and RS co-regulated gene sets described in Fig. 2a, c. Available data was obtained from [16]. The list of PIF-bound genes in each gene set is provided in Supplementary Data 1 and 2.

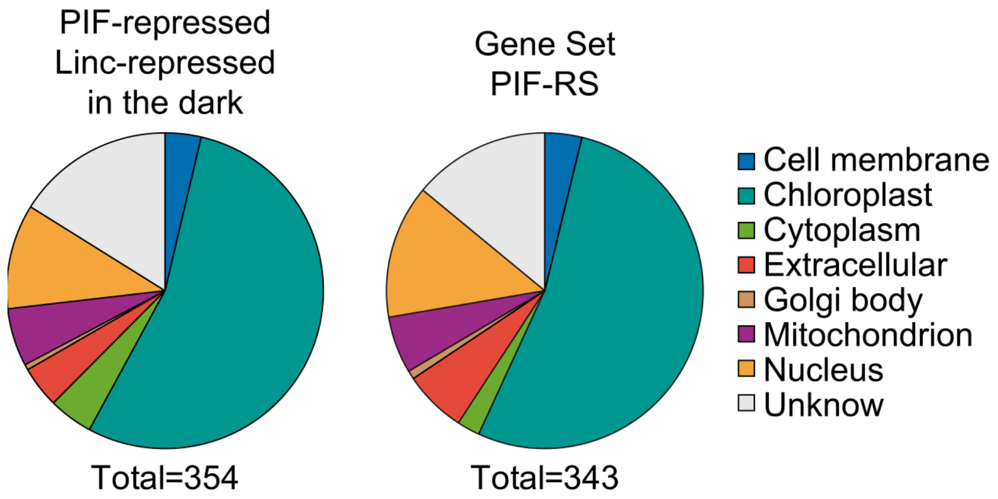

Supplementary Fig. 13. Subcellular localization of PIF- and RS-repressed genes. Subcellular localization of genes in the light/PIF and RS co-regulated gene sets described in Fig. 2a, c, based on Gene Ontology annotations available at TAIR (http://www.Arabidopsis.org). Each class is represented as percentage of the total annotated genes. The list of genes belonging to each class is provided in Supplementary Data 1 and 2.

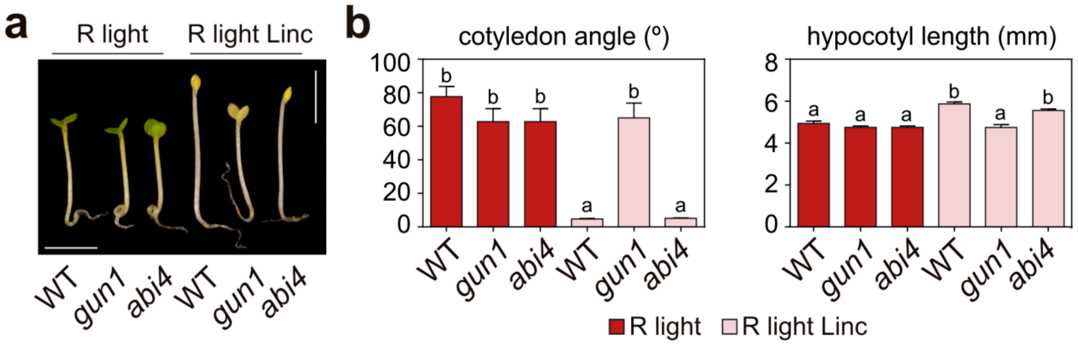

Supplementary Fig. 14. Mutants in *GUN1* partially deetiolate in the presence of lincomycin under continuous red light. (**a**) WT, *abi4*, and *gun1* seedlings were grown for 3 days in continuous red (R) light in the absence (R Light) or presence (R Light Linc) of lincomcyin. Scale bar corresponds to 2.5 mm. (**b**) Cotyledon angle and hypocotyl length of seedlings grown as in (a). Error bars represent SE (n≥20). The experiment was performed twice independently. Different letters denote statistically significant differences among means by Tukey-b's test ($P < 0.05$).

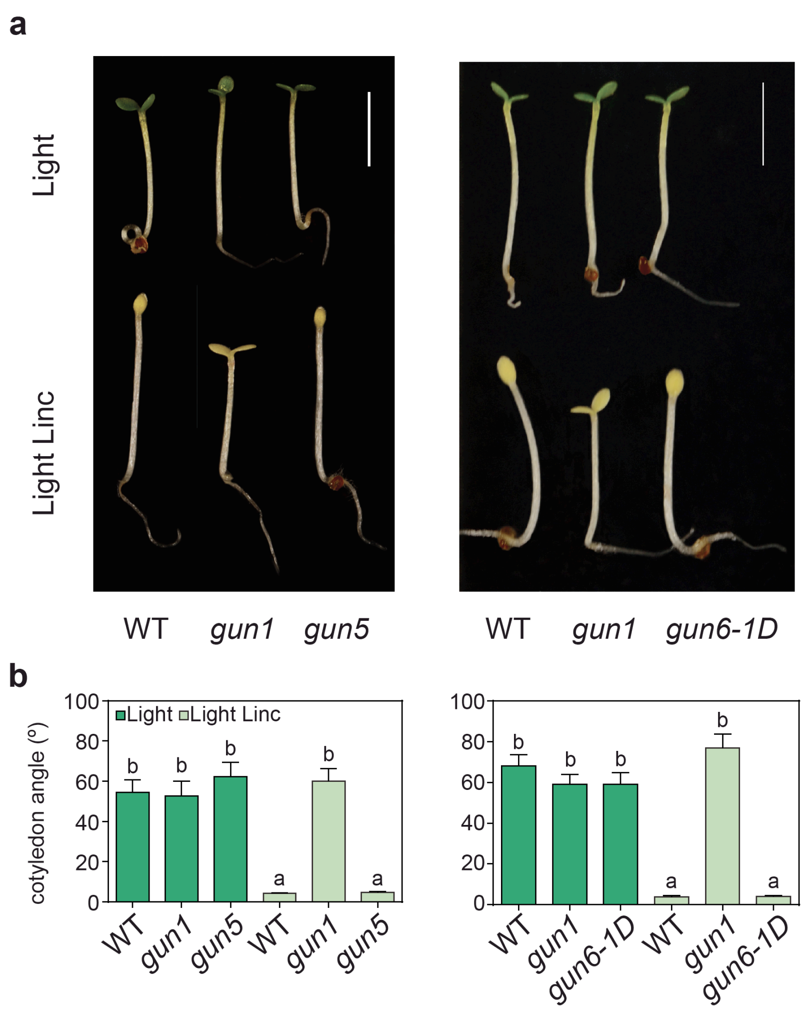

Supplementary Fig. 15. The response of *gun5* and *gun6* mutants to the presence of lincomycin under continuous light is similar to the wild type. (**a**) WT, *gun1*, *gun5* (left) and *gun6-1D* (right) seedlings were grown in continuous white light in the absence (Light) or presence of lincomycin (Light Linc) during three days. Scale bar corresponds to 2.5 mm. (**b**) Cotyledon angle and hypocotyl length of seedlings grown as in (a). Error bars represent SE (n≥25). The experiment was performed independently three times. Different letters denote statistically significant differences among means by Tukey-b's test ($P < 0.05$).

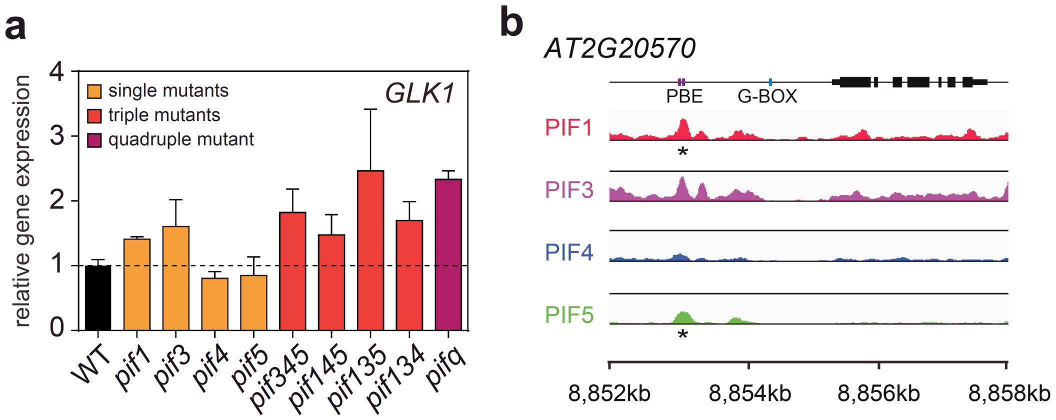

Supplementary Fig. 16. *GLK1* is a PIF-repressed gene directly targeted by the PIFs. Compiled ChIP–seq and RNA–seq data identify *GLK1* as a direct target of PIF transcriptional regulation in the dark. (**a**) Mean expression value of *GLK1* in single *pif1*, *pif3*, *pif4*, and *pif5* mutants, in all the triple *pif* mutant combinations, and in *pifq*. Expression levels are expressed relative to the value of the WT (dashed line). RNA-seq data are from [16]. Data are the means ± SE of biological triplicates. (**b**) Visualization of ChIP-seq data in the genomic region encompassing the *GLK1* locus. Identified significant binding sites are indicated by an asterisk below the pile-up ChIP-seq tracks, and extends to 200bp around the centered peak summit defined as the binding-peak maximum. G- and PBE-box motifs in the promoter are indicated. Data obtained from [16]. *GLK1* was defined as a PIF4-bound gene by ChIP-qPCR [31] and in a ChIP-seq experiment [32].

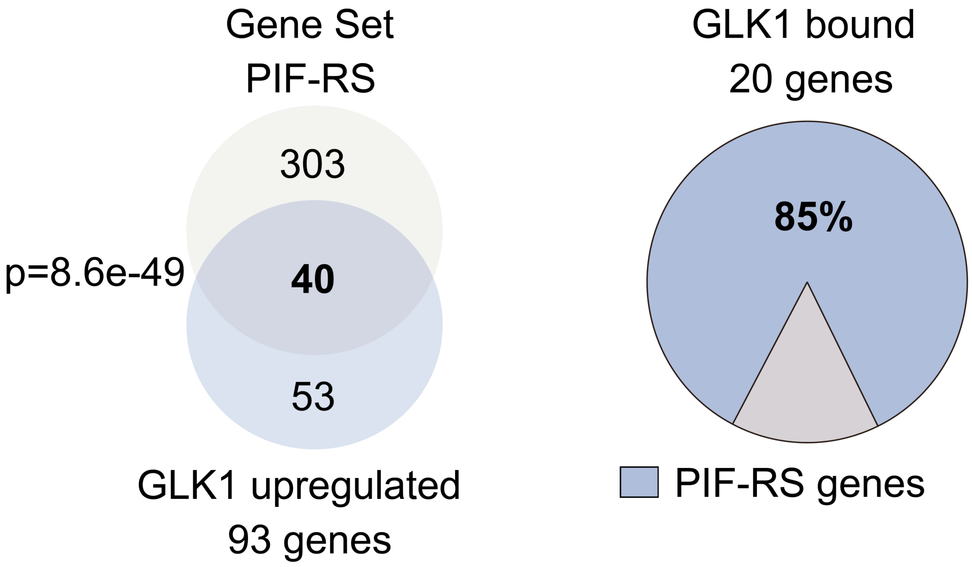

Supplementary Fig. 17. 'Gene Set PIF-RS' is enriched in GLK1-regulated genes. (Left) Venn diagram showing the overlap between genes in gene set PIF-RS and genes upregulated by GLK1 [17] that have a corresponding probe in the ATH1 array. The significance of the number of PIF-RS genes in the GLK1 upregulated gene set compared to the total population of genes represented on the ATH1 array was assessed by the hypergeometric statistical test. (Right) Pie chart showing the percentage of described GLK1-bound genes [17] that are PIF-RS genes.

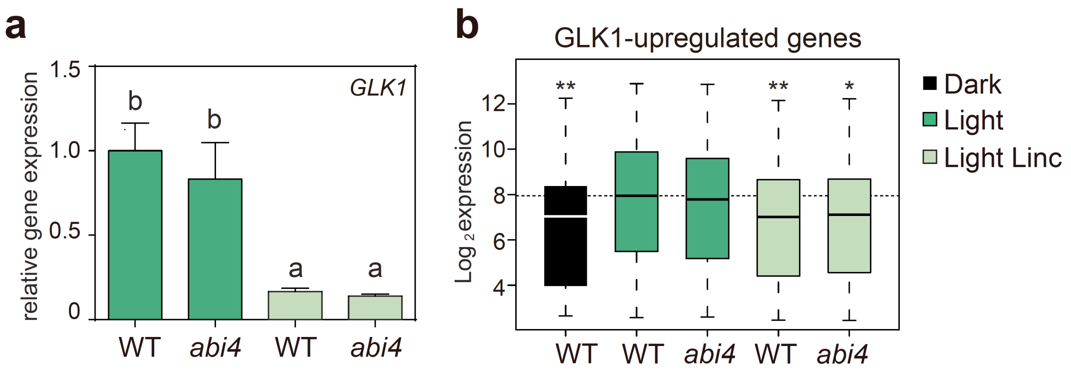

Supplementary Fig. 18. ABI4 does not regulate expression of *GLK1*or GLK1-regulated transcriptional network. (**a**) Expression of *GLK1* analyzed by quantitative RT-PCR in 3-day-old WT and *abi4* seedlings grown in continuous white light in the absence (dark green) or presence (light green) of lincomycin (linc). Values were normalized to *PP2A*, and expression levels are expressed relative to the value of the WT in absence of lincomycin set at one. Data are the means ± SE of biological triplicates. Different letters denote statistically significant differences among means by Tukey-b's test (P < 0.05). (**b**) Expression of GLK1-upregulated genes as defined in [17] in WT and *abi4* in the absence (dark green) and presence (light green) of lincomycin. For comparison, values in dark-grown WT are included (black). The value of the median in light-grown WT in the absence of lincomycin is represented by a grey dashed line. Asterisks indicate statistically significant differences from WT Light by heteroscedastic *t* test (P < 0.05 (*); P < 0.01 (**)). Data obtained from [11, 23].

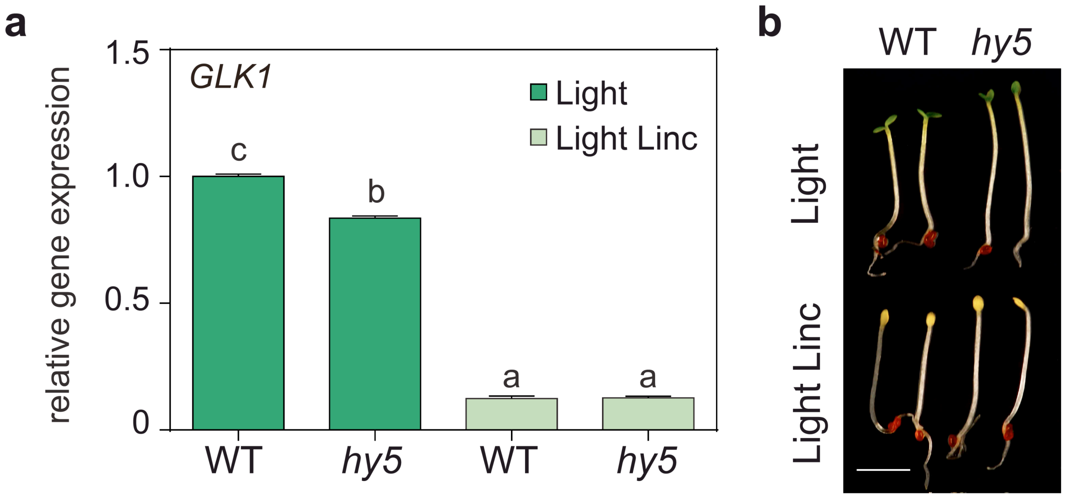

Supplementary Fig. 19. HY5 does not regulate the expression of *GLK1* in response to lincomycin. (**a**) Expression of *GLK1* analyzed by quantitative RT-PCR in 3-day-old WT and *hy5* seedlings grown under continuous white light in the absence (Light) (dark green) or presence (Light Linc) (light green) of lincomycin. Values were normalized to *PP2A*, and expression levels are expressed relative to the value of the WT in absence of lincomycin set at one. Data are the means ± SE of biological triplicates. Different letters denote statistically significant differences among means by Tukey-b's test ($P < 0.05$). (**b**) Visual phenotype of representative seedlings grown as in (a).

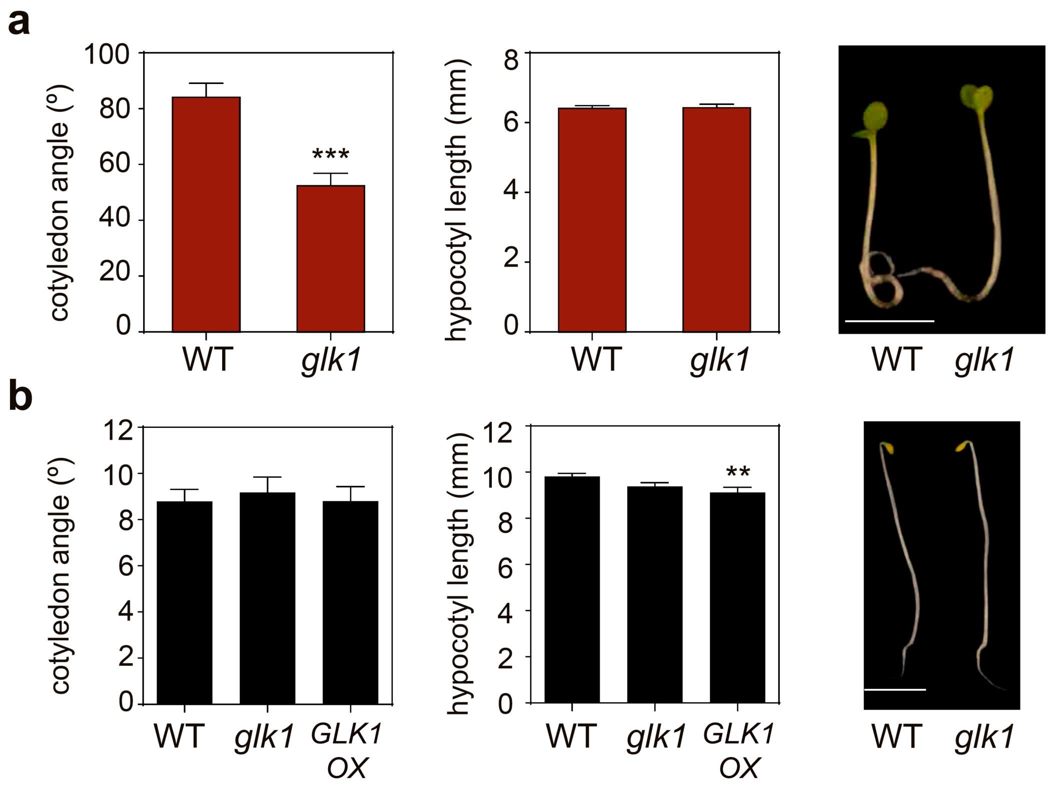

Supplementary Fig. 20. Phenotype of GLK1-deficient (*glk1*) and -overexpressing lines (*GLK1-OX*) under continuous red light and/or dark. (**a**) GLK1 induces cotyledon aperture under continuous red light. Cotyledon angle (left), hypocotyl length (middle), and visual phenotype (right) of 3 day-old wild type (WT) and *glk1* grown under continuous red light.(**b**) *glk1* and *GLK1-OX* display a phenotype similar to WT in the dark. Cotyledon angle (left) and hypocotyl length (middle) of 3 day-old WT, *glk1*, and *GLK1-OX* seedlings grown in the dark. Visual phenotype of 3 day-old WT and *glk1* grown under dark (right). Error bars represent SE of two independent experiments (n≥20). Asterisks indicate statistically significant differences from the corresponding WT by Student's t test (P < 0.01 (**); P < 0.001 (***)). Scale bar represents 2.5 mm.

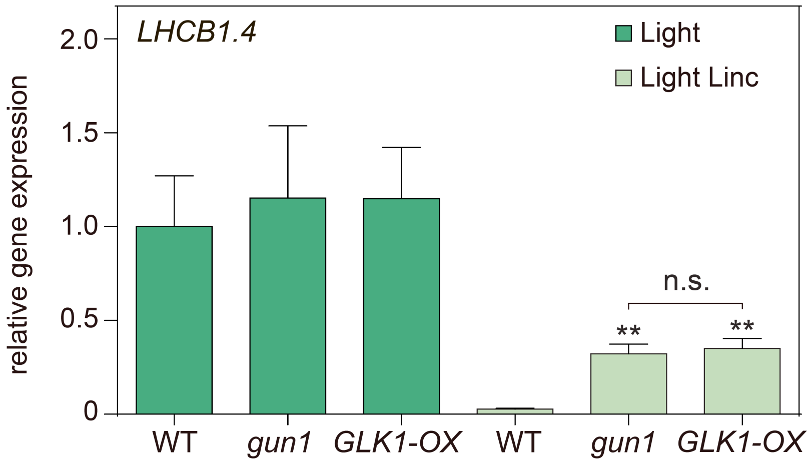

Supplementary Fig. 21. *GLK1-OX* is a *gun* (*genome uncoupled*) mutant in response to lincomycin. Expression of *LHCB1.4* analyzed by quantitative RT-PCR in 3-day-old WT, *gun1*, and *GLK1-OX* seedlings grown under continuous white light in the absence (Light) (dark green) or presence (Light Linc) (light green) of lincomycin. Values were normalized to *PP2A*, and expression levels are expressed relative to the value of the WT in absence of lincomycin set at one. Data are the means ± SE of biological triplicates. Asterisks indicate statistically significant differences by Student's *t* test from their corresponding WT, or comparing *gun1* and *GLK1-OX* Light Linc samples. (P <0.01 (**); n.s., not significant).

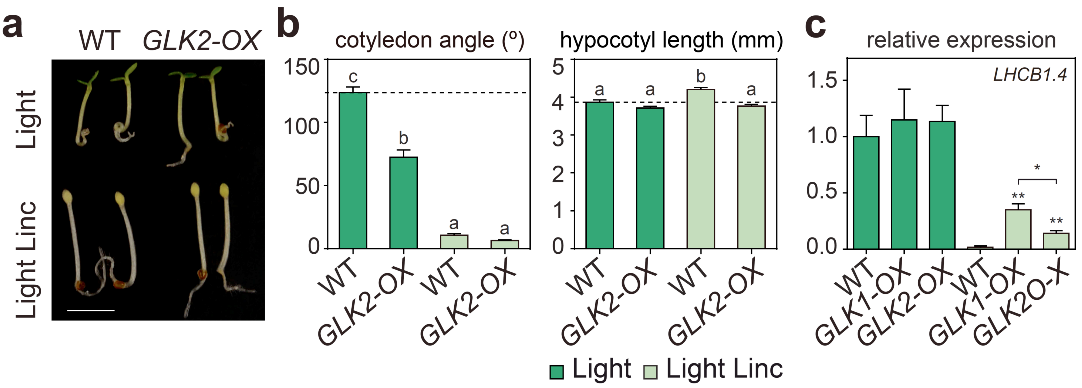

Supplementary Fig. 22. GLK2 regulation of photomorphogenesis in response to lincomycin treatment. (**a**) Light-induced deetiolation of seedlings overexpressing *GLK2* is blocked by lincomycin, similarly to WT and in contrast to *GLK1-OX*. Wild-type and *GLK2-OX* seedlings were grown for 3 days in white light in the absence (Light) or presence (Light Linc) of lincomycin. Scale bar corresponds to 2.5 mm. (**b**) Cotyledon angle and hypocotyl length of seedlings grown as in (a). Error bars represent SE of two independent experiments (n≥30). Different letters denote statistically significant differences among means by Tukey-b's test (P <0.05). The small but significant hypocotyl elongation displayed by WT seedlings in response to lincomycin is absent in *GLK2-OX,* and might indicate that *GLK2-OX* is insensitive to lincomycin for hypocotyl elongation. (**c**) *LHCB1.4* expression is not completely repressed by lincomycinin in *GLK2-OX* compared to WT. Expression of *LHCB1.4* analyzed by quantitative RT-PCR in 3-day-old WT, *GLK1-OX*, and *GLK2-OX* seedlings grown under continuous white light in the absence (Light) (dark green) or presence of lincomycin (Light Linc) (light green). Values were normalized to *PP2A*, and expression levels are expressed relative to the value of the WT in absence of lincomycin set at one. Data are the means ± SE of biological triplicates. Asterisks indicate statistically significant differences by Student's *t* test from their

corresponding WT, or between *GLK1-OX* and *GLK2-OX* Light Linc samples (P <0.05 (*); P <0.01 (**); n.s., not significant).

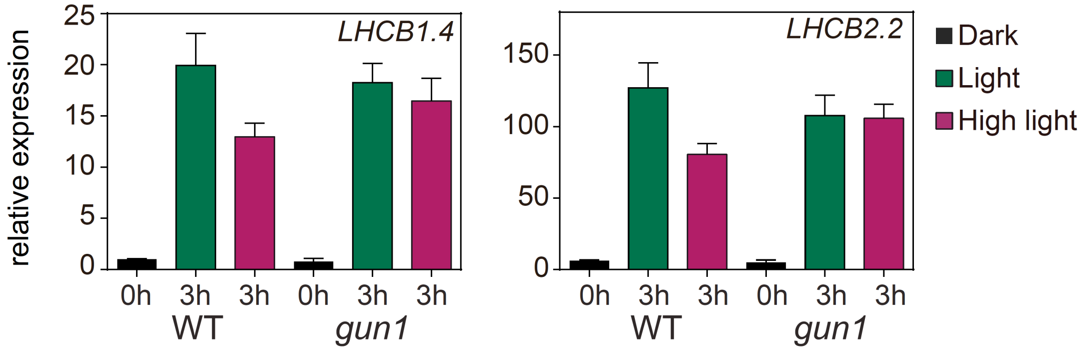

Supplementary Fig. 23. High light triggers GUN1-mediated RS during early seedling deetiolation. Light-induced expression of *LHCB* genes is reduced when seedlings are exposed to High light in a GUN1-dependent manner. Transcript levels of *LHCB1.4* (left) and *LHCB2.2* (right) analyzed by quantitative qRT-PCR in 2-day-old dark-grown WT and *gun1* seedlings transferred for 3 h to white light (Light) (green) or High light (purple). Controls were harvested in the dark at time 0 (black). Values were normalized to *PP2A* and expression levels are expressed relative to WT dark set at one. Data are the means ± SE of biological quadruplicates.

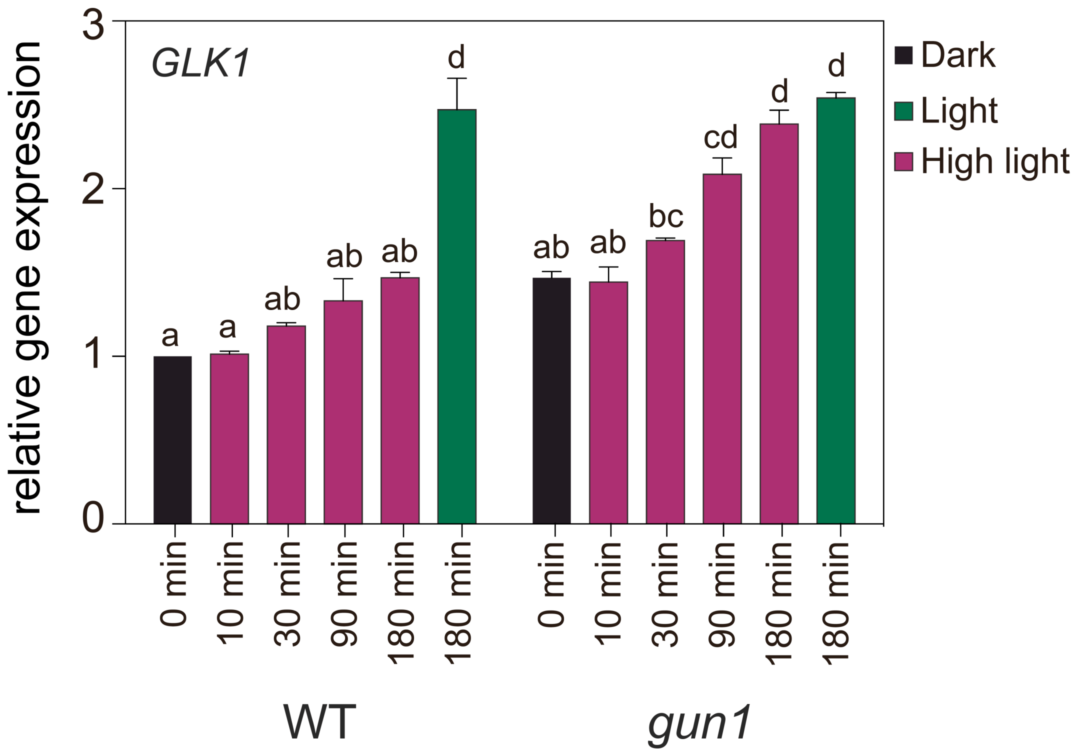

Supplementary Fig. 24. High-intensity light induces GUN1-facilitated repression of *GLK1* expression. Time course induction of *GLK1* expression after 10, 30, 90, and 180 minutes (min) of High light (purple) treatment in 2 day-old dark-grown WT and *gun1* seedlings. Values after 180 min of low Light (green) are shown as reference. Error bars represent SE of two independent experiments (n=3). Different letters denote statistically significant differences among means by Tukey-b's test ($P < 0.05$).

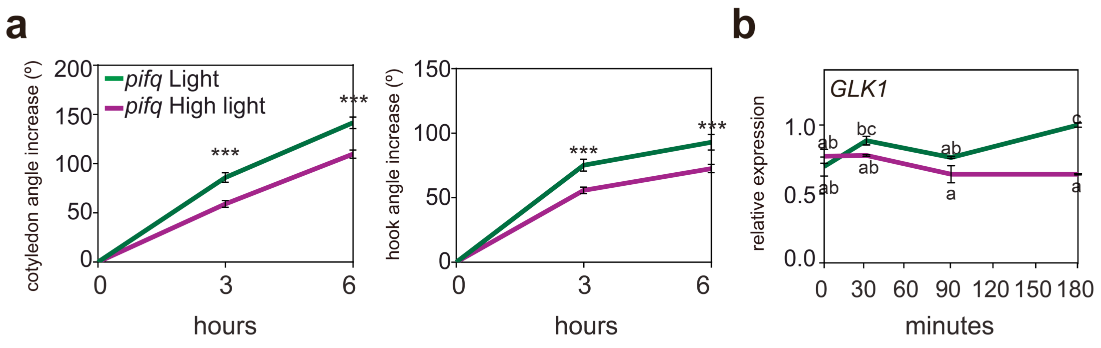

Supplementary Fig. 25. Response kinetics of dark-grown *pifq* seedlings to high light. (**a**) Cotyledon separation (left) and hook unfolding (right) in dark-grown *pifq* seedlings after 3 and 6 hours of High light treatment (purple) compared to low Light (green). Quantification of differences in cotyledon separation (left) and hook aperture (right) was calculated in single seedlings between different time points. Data represent the means ± SE (n≥40). Asterisks indicate statistically significant differences between mean values by Student's *t* test at each time point (P <0.001 (***)). (**b**) Expression of *GLK1* analyzed by quantitative RT-PCR during a 180 minute time course in 2-day-old dark-grown *pifq* seedlings transferred for 3 hours to low white light (Light) (green) or High light (purple). Values were normalized to *PP2A*, and expression levels are expressed relative to the value of the *pifq* grown in the light for 180 minutes. Data are the means ± SE of biological duplicates. Asterisks indicate statistically significant differences by Student's *t* test (P <0.05).

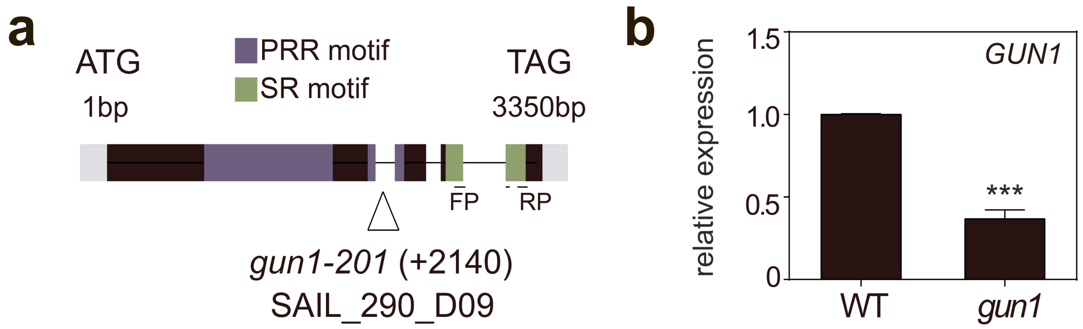

Supplementary Fig. 26. *gun1-201* mutant. (**a**) Map of the *GUN1* gene. The T-DNA insert in *gun1-201* (SAIL_290_D09) is indicated at position +2140 base pairs relative to the ATG. The pentatricopeptide repeat PPR and the small mutS-related domain SMR are indicated as previously described [23]. Position of forward (FP) and reverse (RP) primers used in (b) are also indicated. (**b**) Quantitative RT-PCR analysis of expression of the distal region downstream of the T-DNA insertion of *GUN1* in *gun1-201* using the primers indicated in (a). Values were normalized to *PP2A*, and data are the means ± SE of biological triplicates. Asterisks indicate statistically significant differences by Student's *t* test (P <0.001 (***)).

	SSTF Lincomycin repressed	SSTF Lincomycin induced
Dark-grown WT	161 genes (75%)	73 genes (65%)
Dark-grown *pifq*	384 genes (81%)	581 genes (37%)

Supplementary Fig. 27. Lincomycin-regulated genes in dark-grown WT and *pifq* seedlings. Number of SSTF genes in 3-day-old dark-grown WT (top) and *pifq* (bottom) seedlings regulated by lincomycin as identified by RNA-seq transcriptomic profiling (see Supplementary Note 1). Percentage represents the overlap between Lincomycin-regulated genes that were either repressed (left) or induced (right), excluding genes that are not present in ATH1 array, and the previously reported Retrograde Signaling regulated genes [27]. SSTF: genes whose expression changed statistically significantly and by at least twofold. The list of genes belonging to each class is provided in Supplementary Data 3.

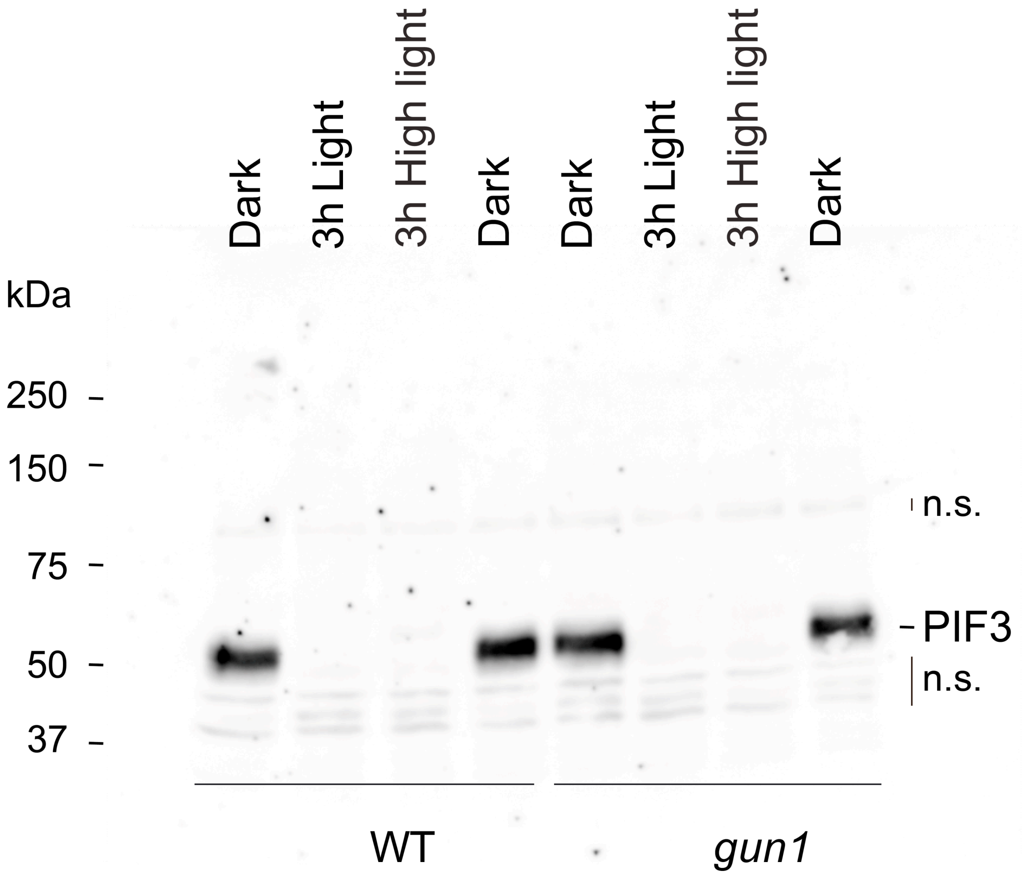

Supplementary Fig. 28. Endogenous PIF3 protein levels in WT and *gun1*. PIF3 protein levels in dark-grown WT and *gun1* seedlings were below level of detection after 3 h in Light or in High light. The uncropped western blot corresponds to the cropped blot shown in Fig. 5e. n.s., non-specific bands.

Supplementary Note 1

RNA-seq transcriptomic analysis of seedlings grown in the presence of lincomycin

We performed RNA-seq analysis of 3 day-old dark-grown *pifq* and WT seedlings grown in presence or absence of lincomycin. First, we compared the lincomycin effect in gene expression in both WT and *pifq* genotypes to define genes statistically significant two-fold (SSTF) regulated in response to lincomycin treatment. In WT seedlings (WT Lincomycin VS WT), we identified 161 SSTF genes repressed by lincomycin and 73 SSTF genes that were induced by lincomycin, and in *pifq* seedlings (*pifq* Lincomycin VS *pifq*) we identified 384 genes repressed by lincomycin whereas 581 were induced (Supplementary Fig. 27). To correlate these changes in nuclear gene expression with the activation of retrograde signaling (RS), we compared these lincomycin-responsive genes with genes described previously as RS-responsive [27]. We observed a large overlap between RS-repressed genes and lincomycin-repressed genes in both WT (75%) and *pifq* (81%), and between RS-induced genes and lincomycin-induced genes in WT dark grown seedlings (65%), whereas only 37% of lincomycin-induced genes in *pifq* overlapped with previously described RS-induced genes (Supplementary Fig. 27). This overlap with previously defined RS-responsive genes indicated that retrograde signaling is activated in our conditions in the dark in the presence of lincomycin.

To further understand RS function in the suppression of the *pifq* photomorphogenic phenotype, we analyzed the transcriptional response of PIF-regulated genes to lincomycin. We compared expression in *pifq* to WT and identified 521 SSTF PIF-repressed and 1826 SSTF PIF-induced genes. Of the 521 SSTF PIF-repressed genes, 354 genes (67.9%) were statistically and significantly (SS) repressed in response to lincomycin in *pifq* mutants (*pifq* VS *pifq* Linc), 38 genes were SS

induced (7.29%), and 129 (24.76%) did not respond to lincomycin (Supplementary Fig. 9). In addition, of the 1826 SSTF PIF-induced genes, 793 genes (43.4%) were SS induced in response to lincomycin, 14 genes were repressed (0.76%), and 1019 (55.80%) were not lincomycin-regulated (Supplementary Fig. 9). These results showed that lincomycin strongly regulates PIF-regulated genes in the same direction, repressing the expression of PIF-repressed genes and inducing the expression of PIF-induced genes. These genes were called 'PIF- repressed reversed' and 'PIF- induced reversed' genes respectively.

Next, we assessed quantitatively the percentage of lincomycin reversion for each of the PIF-regulated reversed genes. To calculate this percentage, we first calculated the lincomycin effect by comparing the difference in expression between dark-grown WT and *pifq* grown in the dark in the presence of lincomycin, with the difference in expression between dark-grown WT and *pifq* grown in the dark in the absence of lincomycin (*pifq* Linc-WT/*pifq*-WT), and applied the formula (1-lincomycin effect) x 100. Values of lincomycin reversion similar to 100% indicate that gene expression in *pifq* dark-lincomycin seedlings was equal to the WT dark values. For the 354 PIF-repressed reversed genes, 331 genes (93%) reverted the expression in response to lincomycin more than 50% toward the dark WT level (3 rightmost panels together in Supplementary Fig. 10a), and 270 genes (76.7%) reverted more than 75% (2 rightmost panels together in Supplementary Fig. 10a). For the 793 PIF-induced reversed genes, only 199 genes (25%) reverted the expression more than 50% (3 rightmost panels together in Supplementary Fig. 10b). These results showed that lincomycin strongly restores the *pifq* transcriptome to the WT dark state. We also conclude that lincomycin had a stronger effect reverting *pifq*-gene expression of PIF-repressed genes, which are overexpressed in *pifq,* than of PIF-induced genes.
